# Waffle‐inspired hydrogel‐based macrodevice for spatially controlled distribution of encapsulated therapeutic microtissues and pro‐angiogenic endothelial cells

**DOI:** 10.1002/btm2.10495

**Published:** 2023-03-14

**Authors:** Chi H. L. Pham, Yicong Zuo, Yang Chen, Nam M. Tran, Dang T. Nguyen, Tram T. Dang

**Affiliations:** ^1^ School of Chemical and Biomedical Engineering Nanyang Technological University (NTU) Singapore Singapore

**Keywords:** diabetes, homogeneous distribution, islets, macro‐encapsulation, microtissue aggregation, vascularization

## Abstract

Macro‐encapsulation systems for delivery of cellular therapeutics in diabetes treatment offer major advantages such as device retrievability and high cell packing density. However, microtissue aggregation and absence of vasculature have been implicated in the inadequate transfer of nutrients and oxygen to the transplanted cellular grafts. Herein, we develop a hydrogel‐based macrodevice to encapsulate therapeutic microtissues positioned in homogeneous spatial distribution to mitigate their aggregation while concurrently supporting an organized intra‐device network of vascular‐inductive cells. Termed Waffle‐inspired Interlocking Macro‐encapsulation (WIM) device, this platform comprises two modules with complementary topography features that fit together in a lock‐and‐key configuration. The waffle‐inspired grid‐like micropattern of the “lock” component effectively entraps insulin‐secreting microtissues in controlled locations while the interlocking design places them in a co‐planar spatial arrangement with close proximity to vascular‐inductive cells. The WIM device co‐laden with INS‐1E microtissues and human umbilical vascular endothelial cells (HUVECs) maintains desirable cellular viability in vitro with the encapsulated microtissues retaining their glucose‐responsive insulin secretion while embedded HUVECs express pro‐angiogenic markers. Furthermore, a subcutaneously implanted alginate‐coated WIM device encapsulating primary rat islets achieves blood glucose control for 2 weeks in chemically induced diabetic mice. Overall, this macrodevice design lays foundation for a cell delivery platform, which has the potential to facilitate nutrients and oxygen transport to therapeutic grafts and thereby might lead to improved disease management outcome.

## 1 INTRODUCTION

Encapsulation of exogenous therapeutic cells in hydrogel‐based devices is a promising strategy enabling long‐term, immunosuppressant‐free cellular therapy for the treatment of hormone and protein‐deficiency diseases, such as type 1 diabetes, hemophilia A, or neurodegenerative diseases.^1^, ^2^ Transplanted cells need to be protected from the host immunological attack while maintaining sufficient metabolic exchange and secretion of their respective therapeutic agents.^3^ The semi‐permeability and high water content of hydrogel facilitate the diffusion‐dependent exchange of nutrients and metabolic wastes between encapsulated cells and their surrounding environment while protecting these therapeutic cells from attack by host antibodies and immune cells.^4^, ^5^

Therapeutic cells or microtissues could be embedded in microencapsulation systems consisting of spherical microcapsules or in macro‐encapsulation systems comprising a variety of shapes and structural designs.^6^ A macro‐device, which consists of a single compartment containing multiple therapeutic microtissues, is removable from the recipient either for cell replenishment or in the event of unexpected complications after transplantation.^7^, ^8^ Thus, macro‐encapsulation devices offer remarkable ease of retrieval over microcapsules, which are often scattered in uncontrolled distribution at the implantation site, rendering it more difficult to retrieve them completely. Moreover, in contrast to the limited design of only spherical geometry for hydrogel microcapsules, macro‐encapsulation systems exploit a wider range of structural designs for the microtissue‐containing compartment. Particularly for islet transplantation, a wide range of designs for diffusion chambers or cell‐laden hydrogel sheets have been evaluated for their immuno‐isolation performance in multiple preclinical studies yielding variable outcomes.^4^, ^8^, ^9^, ^10^ For example, the bi‐layered polytetrafluoroethylene (PTFE) TheraCyte™ macrodevice comprised an inner immuno‐isolating membrane and an outer vascularizing membrane, which were designed to facilitate the ingrowth of new blood vessels to enhance survival of encapsulated islet grafts.^11^ Even though diabetic mice transplanted with islet‐loaded TheraCyte devices remained euglycemic for 6 months,^12^ the hydrophobic PTFE component of these devices elicited macrophage activation and fibrotic reaction in rats.^13^, ^14^

To deliver sufficient tissue dosage for clinically relevant therapeutic efficacy, a macro‐encapsulation device is typically burdened with a high cell packing density, increasing the risk of cell or microtissue aggregation that hampers their viability.^7^ Yang et al. reported decreased viability of microtissues encapsulated in a macrodevice at higher cell loading densities, albeit with the incorporation of convective transport for oxygen and nutrients.^15^ Furthermore, the uncontrolled spatial distribution of therapeutic microtissues in macrodevices also exacerbates the tendency for microtissues to cluster, thus, potentially limiting the mass transfer of oxygen to the encapsulated cells and subsequently resulting in hypoxic cell death as well as an ultimate loss of therapeutic function of the transplanted cells.^16^, ^17^ A multitude of oxygenation strategies such as exogeneous oxygen supply or delivery of angiogenesis agents for vascularization have been explored to tackle the challenge of diffusion‐dependent hypoxia.^18^ However, only a limited number of studies focused on solving the issue of nonhomogenous distribution.^10^, ^19^, ^20^ For example, organized distribution of loaded cells or microtissues in macro‐encapsulation devices might facilitate efficient allocation of nutrients and oxygen, mitigating the need for additional supplies. Specifically, Lee et al. reported an alginate‐collagen composite hydrogel sheet with arrays of homogeneously distributed spheroids for the regulation of blood glucose levels in diabetic mice.^20^ Yet, large spacing between distributed spheroids in this device lowered tissue loading capacity as 11 microtissue‐loaded hydrogel sheets with an area of 1 × 1 cm each were required to restore the glycemic control of one mouse. Transplantation of such large devices not only burdens diabetic recipients but also potentially increases device‐induced host immune reaction due to a higher volumetric ratio of encapsulating materials to the dosage of therapeutic cells.^6^

In addition to the limitation in oxygen diffusion induced by their aggregation due to poor spatial distribution, therapeutic microtissue, or cells also suffer from the inherent lack of vasculature at the implant site surrounding the tissue‐containing device.^21^ In their native biological niche, insulin‐secreting primary islets are supported by rich intra‐islet capillaries in the pancreas, allowing the islets to receive about 10% of the pancreatic blood supply despite these islets constituting only 1% of the mass of this organ.^21^ Therefore, to achieve optimal cellular survival, an encapsulation system should also ideally facilitate the formation of new vascularization in close proximity to the encapsulated cells throughout the device while protecting them from host immune attack. To this end, the incorporation of pro‐angiogenic agents has been explored to promote neovascularization of transplanted therapeutic cells,^21^, ^22^, ^23^ albeit without sufficient spatial guidance to direct the growth pattern of these new blood vessels. As such, the resultant neo‐vasculature could be disorganized, potentially leading to unorderly blood flow and inadequate supply to the therapeutic cells.^24^ Alternatively, a device embedded with a vascular network for the delivery of hepatocyte aggregates was fabricated by photopolymerization of gelatin methacryloyl (GelMA) using a customized stereolithography apparatus for tissue engineering (SLATE).^25^ Although the transplanted cellular graft survived and remained functional 14 days post‐transplantation in mice, the vascular layer in the device was positioned underneath the tissue‐encapsulating layer, potentially impairing diffusion of oxygen and nutrients to the cellular aggregates located at the uppermost plane away from the vascular network.^25^

To address the challenge of microtissue aggregation associated with the requirement for high loading of therapeutic cells, we developed a hydrogel‐based macro‐encapsulation device with micropatterned features directing spatially homogeneous distribution of therapeutic microtissues. We also investigated the feasibility of concurrently incorporating vascular inductive cells for potential establishment of a co‐planar, intra‐device vascular network in close proximity with therapeutic microtissues. The viability and functional characteristics of both therapeutic and vascular‐inductive cell types incorporated in this device were evaluated in vitro with immortalized cell lines. This device, which contained primary rat islets and was reinforced with an additional alginate coating, was also evaluated for its ability to restore glycemic control in chemically induced immunocompetent diabetic mice.

## 2 MATERIALS AND METHODS

### 2.1 Synthesis of GelMA

An amount of 7 g of gelatin (Sigma‐Aldrich) was dissolved by heating in 70 mL of Dulbecco's Phosphate‐Buffered Saline (DPBS) at 50°C. Afterwards, 7 mL of methacrylic anhydride (Sigma‐Aldrich) was added dropwise. After 3 h of reaction, 200 mL of DPBS (Gibco Laboratories) was added to dilute the mixture, and the whole solution was stirred for another 15 min at 50°C. Subsequently, the solution was dialyzed against deionized water in a dialysis tube (Shanghai Rebus Network Technology Co., Ltd.) with a cut‐off molecular weight of 12–14 kDa for 1 week at 37°C. The deionized water was replaced every 2 days. Lastly, solid GelMA was obtained by lyophilization of the dialyzed solution and subsequent storage at room temperature.

### 2.2 Cell culture

Rat insulinoma cell line (INS‐1E cells) was purchased from (AddexBio, USA). INS‐1E cells at passages of 40–70 were used in this work. Cells were cultured in RPMI‐1640 medium (Hyclone) supplemented with 10% heat‐inactivated FBS (Gibco Laboratories), 10 mM HEPES, 1 mM sodium pyruvate, 2 mM L‐glutamine, 100 U mL^−1^ penicillin, 100 μg mL^−1^ streptomycin and 50 μM 2‐mercaptoethanol. Cells were kept in a standard incubator with a 37°C humidified atmosphere of 95% air and 5% CO_2_. Cells were detached at a confluency of ~80% after being treated with 0.25% trypsin–EDTA (Gibco Laboratories) at 37°C for 1 min and replated with a density of ~2 × 10^4^ cells cm^−2^ for subculture. Culture medium was replaced every 3 days.

Primary human umbilical vascular endothelial cells (HUVECs) were purchased from Lonza Bioscience, Singapore. HUVECs at passages of 8–11 were used in this work. Cells were cultured in EGM™‐2 Endothelial Cell Growth Medium‐2 BulletKit™ (Lonza Bioscience Singapore) in 5% CO_2_ at 37°C. At a confluency of ~80%, cells were treated with 0.25% trypsin–EDTA at 37°C for 1.5 min and replated with a density of ~1 × 10^4^ cells cm^−2^ for subculture. Culture medium was replaced every 2 days.

### 2.3 Ex situ fabrication of islet‐like microtissues on agarose micromolds

INS‐1 E microtissues were fabricated using agarose molds as previously described.^26^ First, solid Ultrapure© Agarose (Invitrogen) was autoclaved and dissolved in autoclaved deionized water at 150°C to form a solution with the concentration of 2.3% (w/v). The dissolved agarose solution was pipetted onto silicone templates purchased from 3D Petri dish® (Sigma‐Aldrich) and allowed to solidify at room temperature for 20–25 min. Subsequently, the solidified, micromolded agarose hydrogels were separated from the silicone template using a spatula before being transferred to a 6‐well plate and equilibrated with 3 mL of culture medium overnight prior to cell seeding. Every micromolded agarose hydrogel contained a 16 × 16 array of spheroid‐fabricating recesses with each having a round bottom of 300‐μm diameter and a depth of 800 μm. Before cell seeding, the culture medium in each agarose mold was removed by aspiration. Subsequently, 175 μL of INS‐1E cells suspension at a density of 2.19 × 10^6^ cells mL^−1^ was seeded into each agarose mold and cells were allowed to sink into the recesses for 5 min. Afterwards, 3 mL of fresh culture medium was supplemented to each agarose mold. The seeded cells were allowed to assemble on the agarose mold for 48 hours at 37°C and 5% CO_2_. Afterwards, microtissues from 3 agarose molds were collected and subsequently rinsed 3 times with culture medium and kept in a 6‐well plate prior to fabrication of each WIM device. The images of the collected microtissues were taken under an inverted microscope (Olympus CKX53) at 4× magnification and analyzed using ImageJ.

### 2.4 Fabrication of waffle‐inspired GelMA “lock” component with or without HUVECs

GelMA was patterned to form a waffle‐inspired hydrogel network interspersing square or circular microwells of defined dimension by photolithography (Figure S1). Briefly, 50 μL of 10% (w/v) GelMA prepolymer solution with 0.5% (w/v) I2959 photoinitiator (Sigma‐Aldrich) was deposited onto the lid of a petri dish. Afterwards, this GelMA prepolymer solution was sandwiched in the gap between the lid and a glass slide (Biomedia, Singapore) placed on top of two stacks of spacers each comprising two pieces of coverslips with a total thickness of 150 μm. Subsequently, a photomask with desired micropatterns was placed on the top of the glass slide. Upon exposure to UV light (360–480 nm; 7.9 mW cm^−2^) for 19–25 s, the UV‐exposed portion of GelMA was crosslinked to form the micropatterned network of sidewall with a total area of 1 cm × 1 cm. Afterwards, the glass slide bearing the UV‐crosslinked GelMA pattern was rinsed with DPBS to remove the uncrosslinked GelMA residue that was not exposed to UV light, leaving behind microwells.

In this study, 6 groups of GelMA “lock” patterns were designed with circular and square microwells labeled as C‐200, C‐300, C‐400, S‐200, S‐300, and S‐400 with microwell arrays of 32 × 32, 23 × 23, 18 × 18, 32 × 32, 23 × 23, and 18 × 18, respectively. The label “C” and “S” denoted circular and square microwells, respectively, and the number denoted either the diameter of the circular microwell or the side length of the square microwell. For example, C‐200 is the GelMA “lock” component with circular microwells of diameter 200 μm. The width of the GelMA sidewall separating adjacent microwells for each pattern was designed to be 50 μm.

The bright field images of patterns were taken under an inverted microscope (Olympus CKX53), and the fluorescent images of patterns containing FITC‐Dextran (Sigma‐Aldrich) were taken under an inverted fluorescence microscope (Olympus IX71). The FITC‐Dextran was mixed into GelMA prepolymer solution before the mixture was pipetted onto the petri dish.

For fabrication of each HUVEC‐encapsulating GelMA “lock” component, HUVECs were mixed with GelMA prepolymer solution at a density of 2.5 × 10^7^ cells mL^−1^ before this mixture was dispensed on the lid of the petri dish prior to UV exposure. The HUVECs‐laden GelMA “lock” component was cultured for 7 days before incorporation with of the alginate key encapsulating INS‐1E microtissues.

### 2.5 Fabrication of WIM device encapsulating INS‐1 E microtissues with or without HUVECs

**FIGURE 1:**
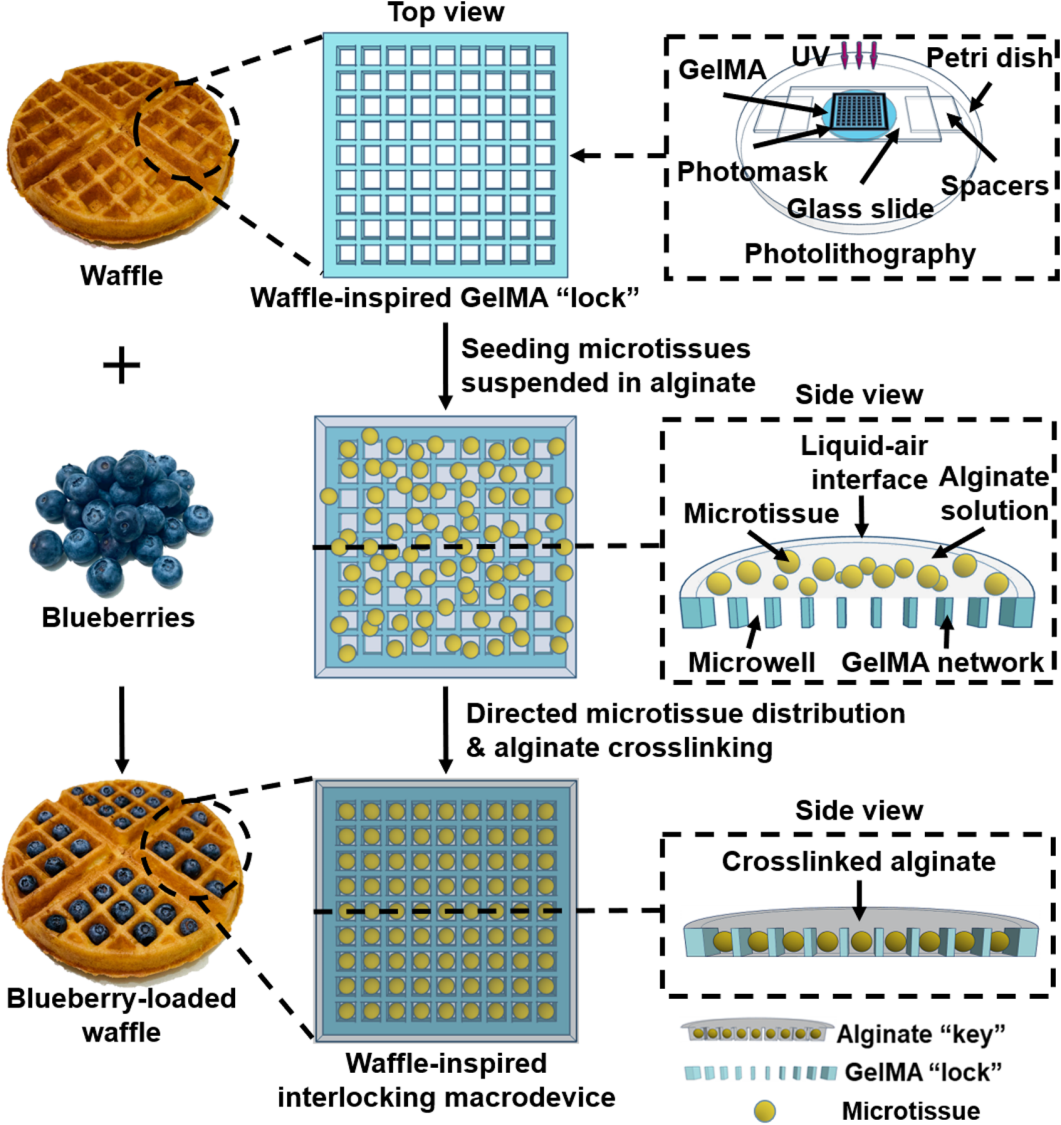
Schematic illustration of the procedure to fabricate Waffle‐inspired Interlocking Macro‐encapsulation device (WIM device). Photolithography was employed to pattern the GelMA “lock” component, followed by addition of a mixture of microtissues and alginate solution, which was subsequently crosslinked to form the alginate “key” component.

Prior to fabricating microtissue‐encapsulating WIM devices, residual DPBS on the GelMA “lock” component fabricated following the procedure described in Section 2.4 was removed by aspiration. Subsequently, 60 μL of 1% (w/v) UP LVG alginate (NovaMatrix) was added on top of the GelMA “lock” component to rinse the surface of the micropatterned GelMA network and kept at room temperature for 30 min prior to removal of this alginate rinsing solution by aspiration. Afterwards, the WIM device was fabricated following a photolithography‐based procedure as illustrated in Figure 1. Approximately 720–750 microtissues collected from 3 agarose molds during ex situ fabrication were suspended in 35 μL of 1% (w/v) UP LVG alginate. The fabricated INS‐1E microtissues have a mean diameter of 146 μm (Figure S2), with each having approximately 1500 cells, estimated by the number of cells seeded per per recess on the agarose mold as described in Section 2.3. The mixture of alginate and microtissues was then dispensed onto the rinsed micropatterned GelMA network and allowed to settle for 10 min. In the next step, 200 μL of 20 mM solution of BaCl_2_ in deionized water was added on top of the dispensed alginate‐microtissue mixture to crosslink the alginate for another 10 min. Following alginate gelation, the whole device was gently detached from the glass slide with a surgical blade and imaged under an inverted microscope (Olympus CKX53). In a parallel experiment, a control device was also fabricated using a similar approach as that used for WIM device. First, a square GelMA frame without the central microwell array was formed on a glass slide using photolithography. Afterward, a mixture of approximately 720–750 microtissues and 35 μL of 1% (w/v) alginate solution was pipetted onto the glass slide within the interior of the GelMA frame followed by addition of BaCl_2_ solution for alginate gelation.

To fabricate WIM device co‐laden with HUVECs and microtissues, HUVECs were mixed with GelMA prepolymer solution at a density of 2.5 × 10^7^ cells mL^−1^ before this mixture was dispensed on the lid of the petri dish prior to UV exposure. The HUVECs‐laden GelMA “lock” component was cultured for 7 days before incorporation with the alginate “key” containing INS‐1E microtissues. Post‐encapsulation live/dead staining, glucose‐stimulated glucose secretion (GSIS) and CD31 immunofluorescent staining were performed after an overnight incubation of 18 h.

### 2.6 Isolation of primary rat islets

This study is conducted under animal protocol A19023 approved by the Institutional Animal Care and Use Committee (IACUC) of Nanyang Technological University (NTU), Singapore. All animal experiments followed the National Advisory Committee for the Laboratory Animal Research (NACLAR), which complies with the National Institutes of Health guide for the care and use of laboratory animals (NIH Publications No. 8023, revised in 1978). Male Sprague–Dawley rats (InVivos Pte Ltd., Singapore) aged 9–10 weeks old were used for islet isolation. A volume of 10 mL of 0.17% (w/v) Liberase (Roche) in cold Hanks' Balanced Salt solution (Sigma) was injected into pancreas of rats through their bile ducts. The fully perfused pancreas was collected and digested for 17–20 min in a 37°C shaking water bath at 120 rpm. Islets were purified by gradient centrifugation using 1100 g/mL Histopaque, prepared with the mixing of 1077 and 1119 g/mL Histopaque (Sigma), followed by six times of sedimentation. Islets were handpicked and cultured in RPMI‐1640 (Hyclone) supplemented with 10% heat‐inactivated FBS (Gibco Laboratories) and 1% penicillin/streptomycin (Gibco Laboratories). Area of islets was measured by ImageJ and effective islet diameter was calculated as the diameter of a circle with the same area.^27^ Based on calculated diameter, islets were converted into islet equivalent (IEQ) using conversion factors as previously described.^28^ Islet circularity was calculated using an ImageJ function based on the formula *Circularity =* $4\pi \times \frac{\text{Area}}{{\text{Perimeter}}^{2}}$,^29^ where area and perimeters were automatically determined by the same function.

### 2.7 Fabrication of alginate‐coated WIM device encapsulating primary rat islets

To fabricate each islet‐encapsulating WIM device following the procedure illustrated in Figure 1, 250 IEQs of primary rat islets were suspended in 30 μL of 1.5% (w/v) SLG20 alginate and dispensed onto S‐300 GelMA “lock” component and followed by alginate crosslinking with a 20 mM BaCl_2_ solution. The whole device was further coated with another layer of alginate (Figure S3). Specifically, the WIM device was detached from the glass slide in Figure S1 and placed on top of a droplet of 200 μL of 2% (w/v) SLG20 alginate which was previously dispensed on another glass slide. The WIM device was then flipped over to expose the other side of the device to the remaining portion of the dispensed alginate. The WIM device with the additional coating of alginate solution was then sandwiched between two Ba^2+^‐enriched agarose hydrogel disks, which were prepared by solidifying a solution of 2.3% (w/v) agarose dissolved in 20 mM BaCl_2_ solution. Subsequently, excess BaCl_2_ solution was added to crosslink the alginate layer on the surface of the sandwiched device for 15 min. Afterwards, the alginate‐coated WIM device was rinsed three times with HEPES buffer and incubated for 4 h in RPMI‐1640 media supplemented with 10% FBS and 1% penicillin–streptomycin for islet recovery prior to transplantation.

### 2.8 Transplantation of WIM device encapsulating primary rat islets into chemically‐induced diabetic mice

Male C57BL/6J mice (InVivos Pte Ltd., Singapore) aged 7–8 weeks old were intraperitoneally injected with streptozotocin (STZ), which was freshly dissolved in 5 mM citrate buffer (Sigma) to form a 7.5 mg/mL solution, at a daily dose of 2.3 mg/30 g for 5‐consecutive days to induce diabetes. Blood glucose level of mice administered with STZ was measured 2 weeks after the first injection of STZ using Accu‐chek Instant S glucose meter (Roche). Induction of diabetes was confirmed if the blood glucose level exceeded 400 mg/dL for three consecutive days and only mice with stable hyperglycemia were used for device transplantation. During subcutaneous transplantation of device, mice were kept anesthetized under 2.5% isoflurane in oxygen. A single incision of 1.5 cm was made at the middle of the back of the mouse and the subcutaneous space was undermined on both sides of the cut. Two alginate‐coated WIM devices containing a total of 500 IEQs (250 IEQs per device) were implanted on the back of each mouse, one on each side of the incision. Following transplantation, blood glucose level of mice was monitored for 2 weeks and value below 200 mg/dL was considered normoglycemia.

### 2.9 In situ formation of microtissues on GelMA “lock” component

Firstly, 80 μL of mono‐dispersed INS‐1E cells suspended in culture medium at a density of 1 × 10^7^ or 5 × 10^6^ cells mL^−1^ was dispensed onto the GelMA “lock” component to achieve a total number of initially seeded cells of 0.8 or 0.4 million, respectively (Figure S11). The seeded cells were subsequently incubated for 4 h at 37°C. After an excess amount of fresh culture medium was supplemented, the sample was incubated for another 20 h and visualized under an inverted microscope. Live/dead fluorescent staining was performed at 24 h post‐seeding.

### 2.10 Visualizing the interlocking of device components

The interlocking of the GelMA “lock” component and alginate “key” component was examined by fluorescent labelling of different components of the WIM device. A suspension of red or green fluorescent polystyrene microbeads (Thermo Fisher) with the diameters of 1 μm were mixed at a concentration of 0.005% (v/v) in the alginate “key” and GelMA “lock” component respectively, while the microtissues were stained with Hoechst dye. Fluorescent images were taken with confocal microscopy (ZEISS LSM 800, Carl Zeiss) and analyzed using ImageJ.

### 2.11 Live/dead fluorescent staining of INS‐1 E microtissues or HUVECs

The viability of INS‐1E microtissues encapsulated in WIM devices in the presence or absence of HUVEC was characterized using LIVE/DEAD™ Viability/Cytotoxicity Kit (Thermo Fisher Scientific). After overnight incubation, the sample on the glass slide was rinsed with 2 mL of 0.9% (w/v) NaCl solution three times. The sample was incubated for 45 min at 37°C in 1 mL of NaCl solution containing 1.6 μM of calcein‐AM and 13 μM of ethidium homodimer‐1. Subsequently, it was rinsed with 2 mL of NaCl solution three times and imaged under confocal microscope (ZEISS LSM 800, Carl Zeiss). Live/dead fluorescent staining of microtissues formed on GelMA “lock” component by in situ assembly of monodispersed INS‐1E cells were similarly performed.

### 2.12 Static GSIS of microtissues encapsulated in WIM device

After an overnight incubation of 18 h following device fabrication, the microtissue‐encapsulating WIM device in the presence or absence of HUVECs was transferred into a 12‐well plate, rinsed three times with 2 mL of 2.8 mM glucose‐contained RPMI‐1640 complete medium, and then pre‐incubated with 1 mL of 2.8 mM glucose‐contained RPMI‐1640 complete medium at 37°C for 1.5 h. Subsequently, the sample was rinsed three times with 2 mL of glucose‐free Krebs Ringer Buffer Hepes (KRBH; 0.5 mM NaH_2_PO_4_, 0.5 mM MgCl_2_, 135 mM NaCl, 5 mM NaHCO_3_, 3.6 mM KCl, 1.5 mM CaCl_2_, 10 mM HEPES and 0.1% (w/v) BSA, pH 7.4). Afterwards, the sample was consecutively subjected to 1 mL of 2.8 mM glucose‐contained KRBH for 1.5 h at 37°C, 1 mL of 16.8 mM glucose‐contained KRBH for 1.5 h at 37°C and 1 mL of 2.8 mM glucose‐contained KRBH for 1.5 h at 37°C. In between two consecutive incubations, the sample was rinsed three times with glucose‐free KRBH buffer. A volume of 100 μL of the supernatant was aspirated at the end of each incubation and stored at −20 °C. Lastly, the insulin concentration in each sample was quantified using Ultrasensitive Insulin ELISA kit (ALPCO Diagnostics).

### 2.13 Immunofluorescent staining of HUVEC‐laden GelMA “lock” component

CD31 expression of HUVECs embedded in GelMA “lock” component was assessed after 7 days of incubation at 37**°**C and 5%CO_2_ following fabrication. Briefly, the sample was consecutively washed in 0.9% (w/v) NaCl three times, fixed in 4% (v/v) formalin solution in NaCl for 30 min, soaked in 0.1% (v/v) Triton X‐100 in NaCl for 30 min and blocked in 10% (v/v) FBS in NaCl for 1 h. Afterwards, a 1:80 dilution of rabbit polyclonal anti‐CD31 primary antibody (Abcam) in 10% (v/v) FBS in NaCl was added and the sample was incubated for 12 h at 4°C. After staining with primary antibody, the sample was washed three times with 0.9% (w/v) NaCl solution with 15‐min intervals in between consecutive washing steps, and subsequently incubated in a 1:200 dilution of Alexa Fluor® 647‐conjugated goat anti‐rabbit secondary antibody (Abcam) in 10% (v/v) FBS in NaCl for 4 h at room temperature. Subsequently, the sample was rinsed six times with 0.9% (w/v) NaCl solution and stained with Fluoroshield Mounting Medium containing DAPI (Abcam) for 5 min. Upon completion of staining, the sample was imaged under confocal microscope (ZEISS LSM 800, Carl Zeiss).

### 2.14 Statistical analysis

All values were averaged and expressed as the mean ± SD. Comparison of insulin secretion following exposure to different glucose concentrations was performed using the one‐way repeated measures ANOVA analysis with Tukey's post hoc test. Comparison of microtissue distribution in C‐200, C‐300, C‐400, S‐200, S‐300, S‐400 WIM devices was performed using one‐way ANOVA with Tukey's post hoc test. A *p*‐value of less than 0.05 was considered statistically significant.

## 3 RESULTS

### 3.1 Structural design of micropatterned macro‐encapsulation device

We developed a dedicated platform to encapsulate therapeutic microtissues with controlled spatial distribution while concurrently supporting an organized intra‐device network of vascular‐inductive cells. Termed **W**affle‐inspired **I**nterlocking **M**acro‐encapsulation (WIM) device, this platform comprised two modules with complementary topography features that fitted together in a lock‐and‐key configuration (Figure 1). Based on a design inspired by the grid‐like pattern on the surface of a waffle, the “lock” component consisted of an interconnected vascular‐supporting hydrogel network, which served as dividing sidewalls separating evenly spaced microwells. The “key” component consisted of therapeutic microtissues encapsulated in a semi‐permeable alginate hydrogel that fitted into the microwells of the “lock” component. We postulated that the waffle‐inspired micropattern of the “lock” component could be designed to direct spatially homogenous distribution of therapeutic microtissues such that each microtissue can be ideally entrapped in one microwell to prevent undesirable aggregation. Furthermore, the interlocking design of our WIM device facilitates the proximity of the vascular‐inductive cells in the “lock” component and the encapsulated therapeutic microtissues in the “key” component.

Figure 1 illustrated a typical procedure to fabricate the WIM device. To fabricate the “lock” component, photolithography was leveraged to design a network of photo‐crosslinked GelMA with desired geometric pattern (Figures 1 and S1) and containing HUVECs, which serve as precursors for potential formation of new blood vessels. An array of uniform empty microwells was left behind after un‐crosslinked region of GelMA was removed. This microfabrication technique has been commonly employed to produce hydrogel microstructures with well‐defined geometry and controlled resolution within the sub‐micron to millimeter range.^30^, ^31^ GelMA was selected as the vascular‐supporting hydrogel due to its ease of photopolymerization and ability to support HUVEC proliferation and organization into tubular structures.^32^, ^33^ Subsequently, the “key” component was fabricated by seeding therapeutic microtissues suspended in an alginate solution on top of the micropatterned “lock” component (Figure 1). Driven by gravity, this liquid mixture filled the microwells of the “lock” component while the microtissues, guided by the GelMA micropattern, also sank and settled into these microwells. Subsequent gelation of alginate by barium ions resulted in the entrapment of these microtissues in alginate hydrogel to form the “key” component. Alginate was selected for encapsulation of microtissues as it has been broadly used as a semi‐permeable membrane for immuno‐protection of cell‐based therapeutics with pre‐clinical success.^34^, ^35^ In the in vitro experiments of this study, insulin‐secreting multi‐cellular spheroids consisting of rat insulinoma INS‐1E cells, which were commonly used to mimic beta cells in the study of type I diabetes, were utilized as the model of islet‐like therapeutic microtissues to be encapsulated in the WIM device. These spheroids were assembled from monodispersed INS‐1E cells prior to their addition onto the GelMA micropattern.^26^ In subsequent preliminary in vivo evaluation, primary islets derived from Sprague Dawley rats were utilized.

### 3.2 Optimizing design parameters of GelMA “lock” component for efficient entrapment and homogenous spatial distribution of microtissues

To the optimize WIM device for efficient entrapment and homogenous distribution of islet‐like microtissues, we fabricated several designs of its GelMA “lock” component by varying its micropattern to yield microwells with different shapes and sizes (Figure S4 **)**. Square and circular microwell shapes were chosen due to their simple geometries, which facilitated their microfabrication with high fidelity from photomask designs. Microwell dimensions, specifically the side‐length of square microwells and the diameter of circular microwells, at 200, 300, and 400 μm were evaluated. The lower limit of microwell dimension was specified as 200 μm to sufficiently accommodate potential therapeutic microtissues, such as rodent and human islets, the mean diameters of which range from 100 to 150 μm.^36^ The upper limit of microwell size was set at 400 μm to ensure a distance of at most 200 μm between cells in the microwell and the vascular inductive cells in the surrounding GelMA sidewalls. This maximum distance of 200 μm has been postulated as the critical diffusion limit between cells and surrounding blood vessels to ensure adequate supply of oxygen, nutrients, and metabolites.^37^ Ex situ fabricated INS‐1E microtissues with a mean diameter of 146 μm (Figure S2), similar to the average dimension of human islets,^38^ were encapsulated in the “key” component of each WIM device.

**FIGURE 2:**
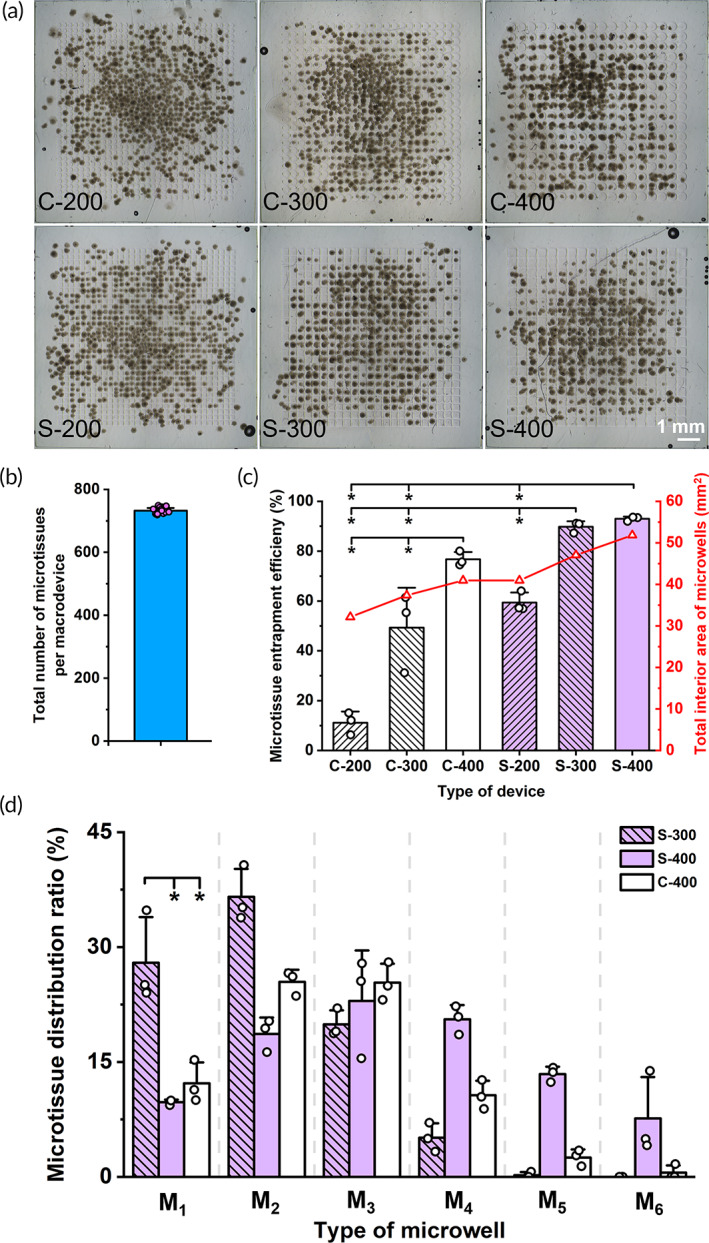
Entrapment efficiency and spatial distribution of microtissues in WIM devices with different designs of GelMA “lock” component. (a) Representative bright‐field images of WIM devices with different designs of GelMA “lock” component immediately after encapsulation of INS‐1E microtissues. The label “C” and “S” denoted circular and square microwells respectively and the number denoted either the diameter of the circular microwell or the side length of the square microwell. For example, C‐200 is GelMA “lock” component with circular microwells of diameter 200 μm. Scale bar representing 1 mm applies to all images in panel A. (b) Total final number of encapsulated microtissues per WIM device (1 × 1 cm) (*n* = 18 devices from 3 batches of devices which were also used to obtain data for C and D) as determined by manual counting of encapsulated microtissues in the images similar to those used for panel A. **(**c**)** Quantitative analysis of microtissue entrapment efficiency and total microwell area for each type of WIM device (*N* = 3 independent experiments). Microtissue entrapment efficiency is defined as the ratio of the total number of microtissues each effectively entrapped within the interior a microwell to the total number of microtissues encapsulated in the entire WIM device. (d) Quantitative analysis of microtissue distribution ratio in S‐300, S‐400, and C‐400 WIM devices. Microtissue distribution ratio is defined as the ratio of the combined number of microtissues in all microwells of type Mn, where *n* is the number of microtissue(s) per microwell and ranges from 1 to 6, to the total number of microtissues encapsulated in the entire macrodevice (*N* = 3 independent experiments). (*) denotes statistical difference (*p* < 0.05). Data are shown as mean ± SD.

As shown in Figure 2a, the micropattern of six WIM devices with different designs of GelMA “lock” components were preserved post‐fabrication without visible deformation or breakage. A composite bright field image of each device, compiled by merging images of different parts of the device, was examined to determine the total number of microtissues encapsulated in each device including the microtissues entrapped within the microwells and those remaining on the GelMA sidewalls or at the outer edge of the device. An initial total number of microtissues of about 720–750 were suspended in alginate and seeded on each GelMA “lock” component. As shown in Figure 2b, above 90% of microtissues were eventually encapsulated in each of the six device designs indicating minimal microtissue wastage.

#### 3.2.1 Effect of microwell design on entrapment of microtissues in GelMA “lock” component

Minimal microtissue aggregation within WIM device is associated with the effective entrapment or confinement of microtissues within the interior space of each microwell while minimizing microtissue positioning on the microwell sidewall to decrease potential contact between microtissues from adjacent microwells. In this experiment, microtissue is deemed as effectively entrapped in a microwell if at least half of its volume is confined within the microwell (Figure S5). For each “lock” design, we quantitatively evaluated its microtissue entrapment efficiency, which is defined as the ratio of the total number of microtissues each effectively entrapped within the interior of a microwell to the total number of microtissues encapsulated in the entire device. A higher microtissue entrapment efficiency was more desirable as fewer microtissues settled on top of the GelMA sidewall or outer edges of the device. Figure 2c shows that for the same device size (1 × 1 cm), a larger total interior area of microwells per device type generally correlated with a higher microtissue entrapment efficiency. For the same microwell shape of either circular geometry (C‐200, C‐300, and C‐400) or square geometry (S‐200, S‐300, and S‐400), the larger microwell size correlated with a higher entrapment efficiency. In addition, square microwell design resulted in a higher entrapment efficiency of the “lock” component compared to that of “lock” component designs with circular microwells, which had the same diameter as the side‐length of the square microwells. Specifically, S‐300 and S‐400 devices had the highest microtissue entrapment efficiency of approximately 90% due to their largest total microwell areas. The microwells in these two devices only failed to entrap about 10% of seeded microtissues, which landed on either the GelMA sidewall or the outer edge of the WIM devices. In contrast, C‐200 device had the lowest entrapment efficiency of only ~10% due to its smallest total microwell area. Interestingly, C‐400 device was able to entrap microtissues more efficiently (~76%) than S‐200 device (~55%) despite their similar total microwell areas. This was possibly attributable to the bigger size of individual 400 μm‐diameter microwells in the C‐400 WIM devices, which were able to better accommodate INS‐1E microtissues with diameter of about 150 μm.

#### 3.2.2 Effects of device design parameters on spatial homogeneity of microtissue distribution

In Figure 2d, we quantified the microtissue distribution ratio in S‐300, S‐400, and C‐400 WIM devices which were the three devices with the highest microtissue entrapment efficiencies of at least 75% from Figure 2c. This microtissue distribution ratio is defined as the ratio of the combined number of microtissues in all microwells of type *M*_*n*_, where *n* is the number of microtissue(s) for a particular microwell and ranges from 1 to 6, to the total number of microtissues encapsulated in the entire WIM device. Since a spatial distribution with one microtissue entrapped in each microwell is considered ideal, a high microtissue distribution ratio for microwells of type M_1_ is considered optimal. About 18%–25% of the microwells in each device remained unoccupied (Figure S6) while the remaining microwells in the same device receive a range of 1 to 6 microtissues in each microwell. Among the three WIM devices evaluated (Figure 2d), S‐300 WIM device presented a higher microtissue distribution ratio for microwells of type M_1_ compared to the other two devices so this design was chosen for further experiments.

### 3.3 Characterization of structural components of WIM device

**FIGURE 3:**
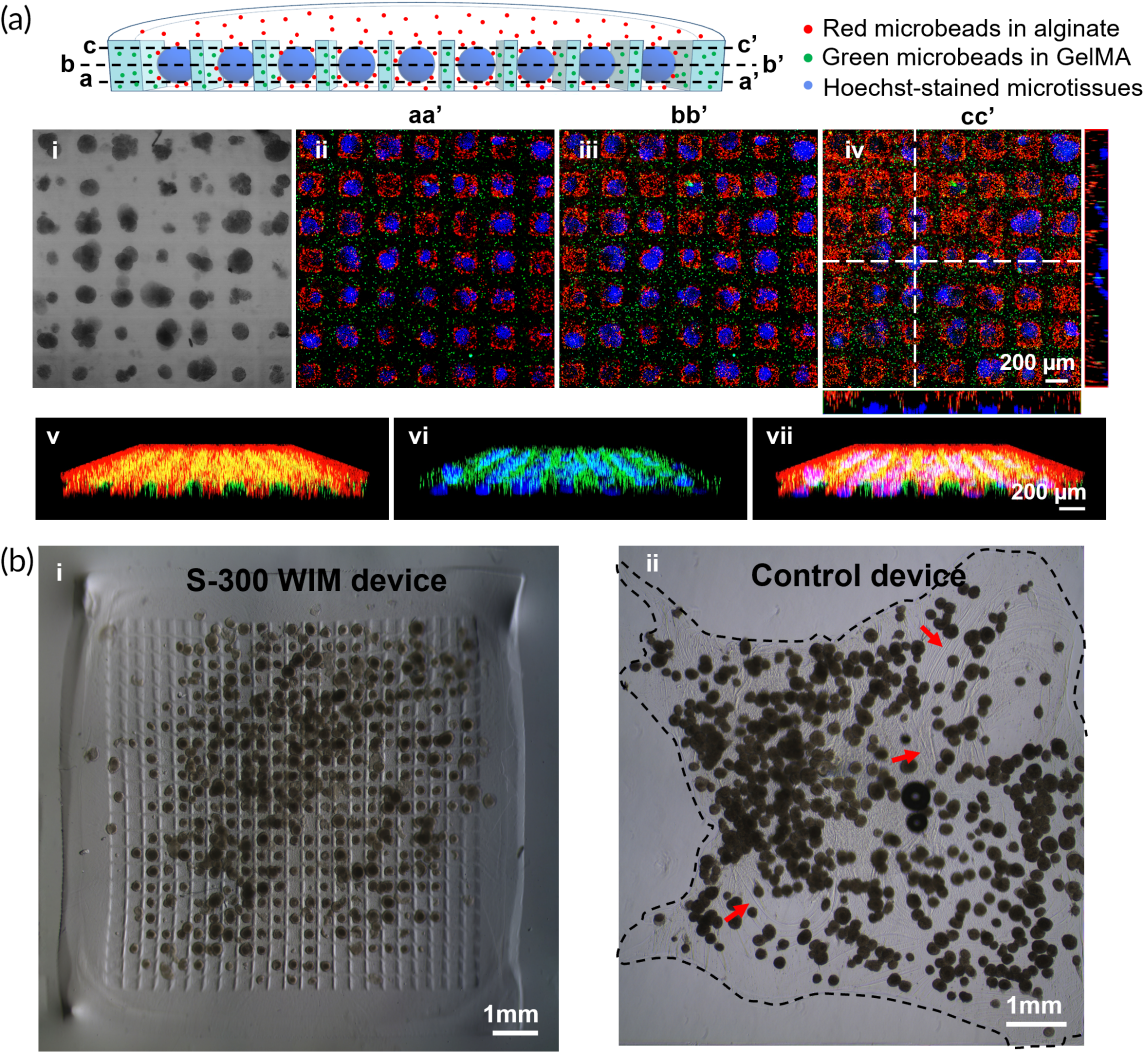
Characterization of structural components of S‐300 WIM device. (a) Interlocking components of WIM device visualized using fluorescence‐emitting polystyrene microbeads. Green fluorescent microbeads were mixed in GelMA, and red fluorescent microbeads were mixed in alginate. INS‐1E microtissues were stained with Hoechst dye emitting blue fluorescence. Bright‐field image of S‐300 WIM device (i) and confocal cross sections of WIM device, that are, (ii), (iii), (iv) at three vertical heights corresponding to lower (*aa*′), middle (*bb*′) and upper (*cc*′) planes of microwells respectively. Scale bar representing 200 μm in image a (iv) applies to a (i–iii). Snapshots of three‐dimensional (3D) views of S‐300 WIM device showing merged red and green channels (v), merged blue and green channels (vi) and merged blue, red and green channels (vii). Scale bar representing 200 μm in image A (vii) applies to A (v–vi). (b) Bright‐filed images acquired immediately after device fabrication of the S‐300 WIM device (i) and the control macrodevice (ii) which did not have a GelMA, that is, “lock” component. Red arrows indicate wrinkled area of control macrodevice.e. Scale bars: 1 mm.

To examine the structural interlocking between the GelMA “lock” component and the alginate “key” component, we labeled each component of the S‐300 WIM device with fluorescent microbeads and visualize them by confocal imaging at three cross sections of the device at three different heights, namely the lower (aa′), middle (bb′), and upper (cc′) planes of the microwells (Figure 3a). Specifically, alginate “key” and GelMA “lock” components were labeled by mixing their precursor solutions with red and green fluorescent microbeads, respectively, whereas cellular nuclei of INS‐1E microtissues were stained with Hoechst dye emitting blue fluorescence. At all three different depths (***aa*** ′, ***bb*** ′, and ***cc*** ′), the central circular regions with blue signal, each surrounded by a thin layer of red signal, were separated by a grid‐like network of green signal (Figure 3a (ii–iv)). This observation demonstrated that alginate‐coated microtissues were separated by GelMA sidewalls and could settle vertically into the interior of individual microwells. Furthermore, each microwell of this WIM device accommodated at most a few microtissues thus minimizing their clumping into undesirable large aggregates and facilitating better controlled and homogeneous spatial distribution of individual microtissues. The 3D view snapshots of the S‐300 WIM device (Figure 3a **(**v–vii)) also showed that blue‐fluorescent regions, each surrounded by a thin layer of red signal, alternated with green fluorescent sidewalls, thus, demonstrating the coplanar interlocking of microtissues surrounded by alginate with GelMA sidewalls.

After the alginate “key” component was fully crosslinked with BaCl_2_ solution, the structural integrity of the S‐300 WIM device was examined with bright field microscopy following its detachment from the glass slide. Figure 3b showed that microtissues encapsulated in S‐300 WIM device exhibited higher homogeneity of spatial distribution while a similar number of microtissues embedded in the control device exhibited a tendency to cluster. Interestingly, the detached S‐300 WIM device maintained its original configuration with minimal alteration in the structural appearance of the GelMA “lock” component (Figure 3b(i)). Furthermore, the WIM device retained its structural integrity without any observed fusion of encapsulated microtissues after 2 days post‐fabrication (Figure S7). In contrast, the control device comprising microtissues encapsulated in a monolithic alginate sheet exhibited microtissue aggregation together with wrinkled features indicated at by red arrows on Figure 3b(ii).

### 3.4 Evaluation of cellular viability and function of encapsulated microtissues

Preservation of cellular viability is a critical criterion influencing the functional performance of the encapsulated therapeutic cells. Thus, we examined the viability of INS‐1E microtissues encapsulated in the S‐300 WIM device by staining them with Calcein‐AM and ethidium homodimer‐1 followed by confocal microscopy to identify live and dead cells by green and red fluorescence, respectively.^26^

**FIGURE 4:**
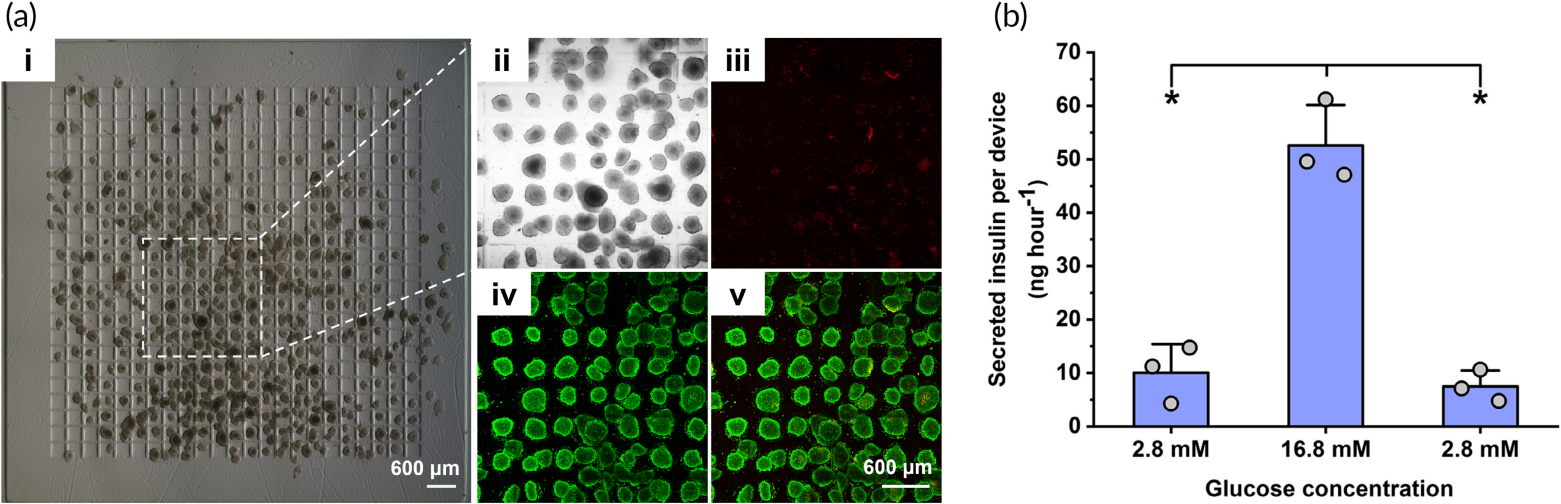
In vitro viability and glucose‐responsive insulin‐secreting function of INS‐1E microtissues encapsulated in S‐300 WIM devices (a) Bright‐field and confocal images of encapsulated microtissues at the same region of interest after live/dead staining. Green fluorescence confirmed that most cells in microtissues were viable while red fluorescence representing cell death was negligible. Images were acquired 18 h after device fabrication. Scale bar representing 600 μm in image a (v) applies to a (ii–iv). (b) Static glucose stimulated insulin secretion of microtissues during consecutively incubations in different glucose concentration for 1 h at each concentration. Data are shown as mean ± SD (*n* = 3 replicate samples). Data are representative of *N* = 2 independent experiments. (*) denotes statistical difference (*p* < 0.05).

Figure 4a(i) showed a bright field image of the stained S‐300 WIM device encapsulating INS‐1E microtissues after overnight incubation. A region of interest (Figure 4a(ii)) on this device was subsequently examined at higher magnification with confocal microscopy. Specifically, Figure 4a(iii–v) each showed the combined projected images of the confocal Z‐stack acquired at the same region of interest on this S‐300 WIM device and visualized with different fluorescent channels. All microtissues appeared dominantly green in the green channel (Figure 4a (iv)) and merged image (Figure 4a(v)), demonstrating that most cells on the surface of these microtissues remained viable. Minimal number of dead cells was concurrently visualized with red fluorescence (Figure 4a(iii)), further confirming negligible cell death observed for these encapsulated microtissues.

A successful encapsulation strategy for therapeutic cells allows not only the preservation of cellular viability but also their desired function. Therapeutic cells after encapsulation must still secrete insulin in an appropriate amount according to physiological glucose levels, because both excessive or inadequate insulin secretion can result in abnormal glycemia in diabetic recipients.^39^ Microtissue‐encapsulating S‐300 WIM device was subjected to the static GSIS assay to evaluate the insulin‐secreting function of encapsulated INS‐1E microtissues. A sequential incubation of the device at 2.8, 16.8, and 2.8 mM glucose levels simulated the physiological condition in diabetic patients, alternating from basal condition to hyperglycemia and back to basal condition respectively. As shown in Figure 4b and Figure S8, the amount of insulin secreted by the encapsulated microtissues increased 6.6‐fold when they were exposed to the higher level of glucose in the second incubation compared to the first incubation at a lower glucose level. This increase was comparable to the outcome from published studies investigating the responsive insulin secretion of INS‐1E spheroids.^40^ During third incubation, the microtissues exhibited a return to basal insulin secretion, indicating their preserved function and responsiveness.

### 3.5 Preliminary in vivo evaluation of S‐300 WIM device function containing primary rat islets in chemically induced diabetic mice

**FIGURE 5:**
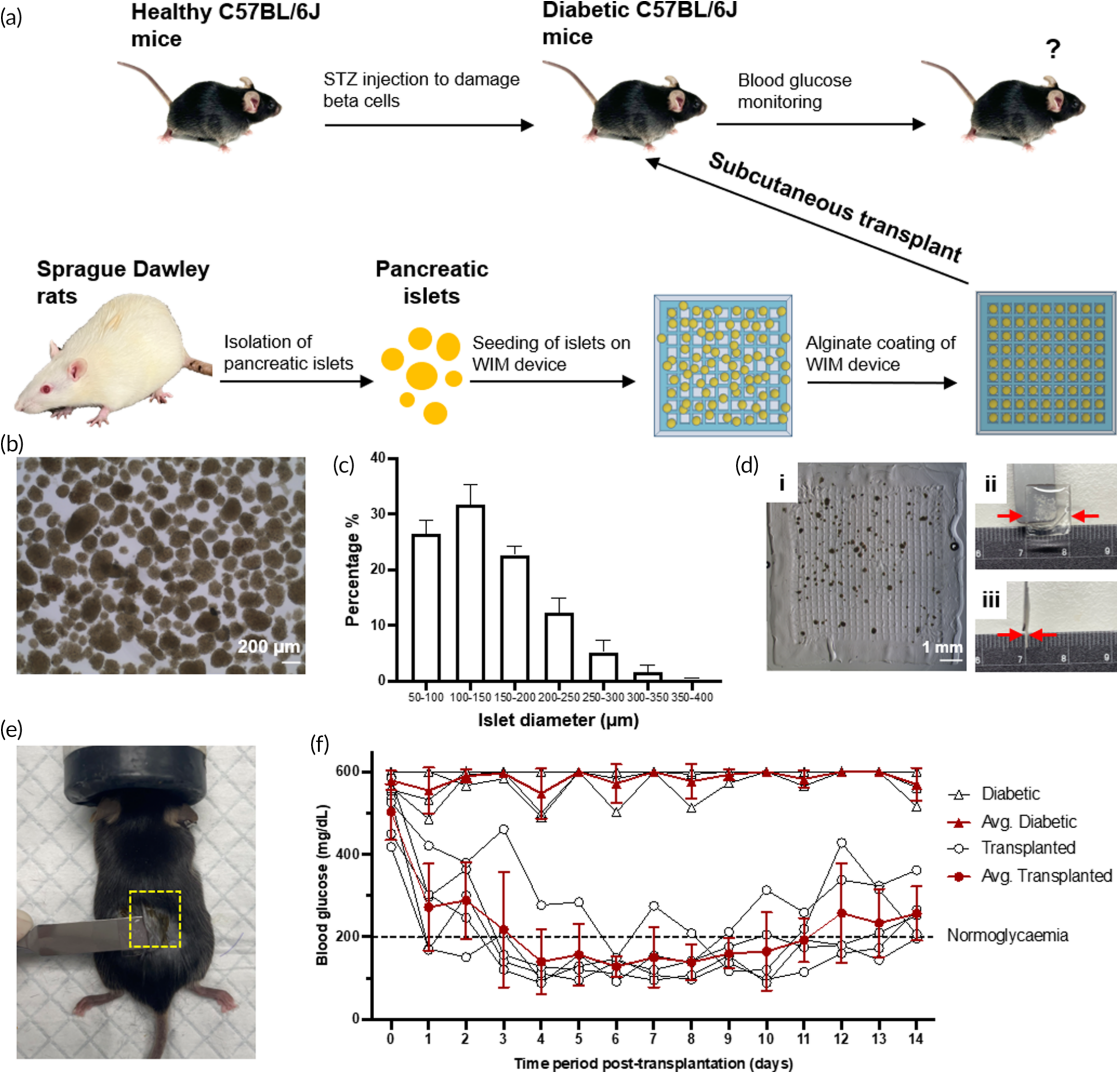
In vivo glycemic correction function of S‐300 WIM device encapsulating primary rat islets in STZ‐induced diabetic C57BL/6J mice. (a) Schematic procedure for evaluation of S‐300 WIM device containing primary rat islets for subcutaneous transplantation into chemically induced diabetic C57BL/6J mice. (b) Bright‐field image of primary rat islets from Sprague Dawley rats immediately after isolation. Scale bar: 200 μm. (c) Size distribution of primary islets based on their diameters (*N* = 6 batches of islet isolation, each batch of 3–6 rats). (d) Bright‐field image of S‐300 WIM device encapsulating primary rat islets (i) and photographs of its side (ii) and front view (iii). Scale bar represents 1 mm for (i). (e) Subcutaneous transplantation of device on dorsal side of a diabetic mouse. (f) Daily nonfasting blood glucose concentration of STZ‐induced diabetic mice transplanted with S‐300 WIM devices (*n* = 5) containing primary rat islets and untreated diabetic mice (*n* = 4). Data are shown as mean ± SD.

Preservation of cell survival and insulin‐secreting function in vivo for glycemic control is an important criterion towards desired treatment efficacy of a cell delivery strategy. The efficacy of S‐300 WIM device in delivering insulin‐secreting cells for glycemic correction was evaluated in a chemically induced mouse model of type I diabetes. Specifically, we evaluated in a preliminary experiment whether the dimension of the S‐300 WIM device allowed sufficient release of insulin in vivo to restore glycemic control. Two S‐300 WIM devices were loaded with a total 500 IEQs of primary islets isolated from Sprague Dawley rats prior to its subcutaneous transplantation on the dorsal side of each Streptozotocin‐induced diabetic C57BL/6J mouse, which was monitored for blood glucose level over 14 days (Figure 5a).

We examined the size and shape of isolated islets prior to their encapsulation in the S‐300 WIM device. Rat islets had a wide range of size distribution from 50 to 400 μm in diameter (Figure 5b, c), with most islets having diameters within the range of 50–200 μm. The most frequently occurring islet diameter was between 100 and 150 μm (Figure 5c), which is similar to the average dimension of engineered INS‐1E microtissues obtained from ex situ fabrication (Figure S2). Nonetheless, primary rat islets were more heterogeneous in shape as measured by their circularity (Figure S9). This parameter, defined as the ratio of area to squared perimeter of an islet, was used to quantify the resemblance of the two‐dimensional (2D) projection of islet geometry to an ideal circle.^36^ In our experiment, primary rat islets were of significantly lower level of circularity (0.76) in contrast to that of uniform INS‐1E microtissues (0.85) (Figure S9), hence probably rendering it more difficult to achieve the same level of homogeneous spatial distribution for the islets in the S‐300 WIM device. After gelation of the alginate “key,” the device was further coated (Figure S3) in another layer of alginate that covered the whole device to increase its mechanical strength (Figure S10) for subcutaneous transplantation. The alginate‐coated S‐300 WIM device preserved the interlocking feature (Figure 5D(i)) with adequate thickness (Figure 5d(iii)) for sufficient release of therapeutic insulin from the device to achieve glycemic correction over 2 weeks. The final alginate‐coated S‐300 WIM device with a dimension of 1 × 1 × 0.1 cm (Figure 5d) allowed simultaneously transplantation of two devices containing a total of 500 IEQs in the dorsal region of each mouse (Figure 5e). During the two‐week experiment, the transplanted alginate‐coated S‐300 WIM devices restored and maintained normoglycemia in diabetic mice (Figure 5f).

### 3.6 Assembly of monodispersed cells for in situ formation of microtissues with homogenous spatial distribution

Islet‐like microtissues or pseudo‐islets, which are engineered microtissues assembled in situ or “on device” from mono‐dispersed primary islet‐derived cells, might be used in place of irregularly shaped primary rat islets.^41^, ^42^ In situ fabrication of microtissues comprises the initial step of microtissue assembly on one platform and the subsequent step of encapsulating microtissues on the same platform with semi‐permeable hydrogel to form the final macroencapsulation device.^20^ In contrast, ex situ or “off device” fabrication of microtissues involve assembly of microtissues on another platform prior to transferring them onto a second platform to be encapsulated in semi‐permeable hydrogel.^26^ Hence, in situ microtissue fabrication eliminates the risk of cell mass loss associated with microtissue transfer and replating between the two platforms used in ex situ fabrication approach. Therefore, we evaluated the feasibility of fabricating the S‐300 WIM device using an in situ approach for fabrication of islet‐like microtissues on GelMA “lock” component (Figure **S11**).

**FIGURE 6:**
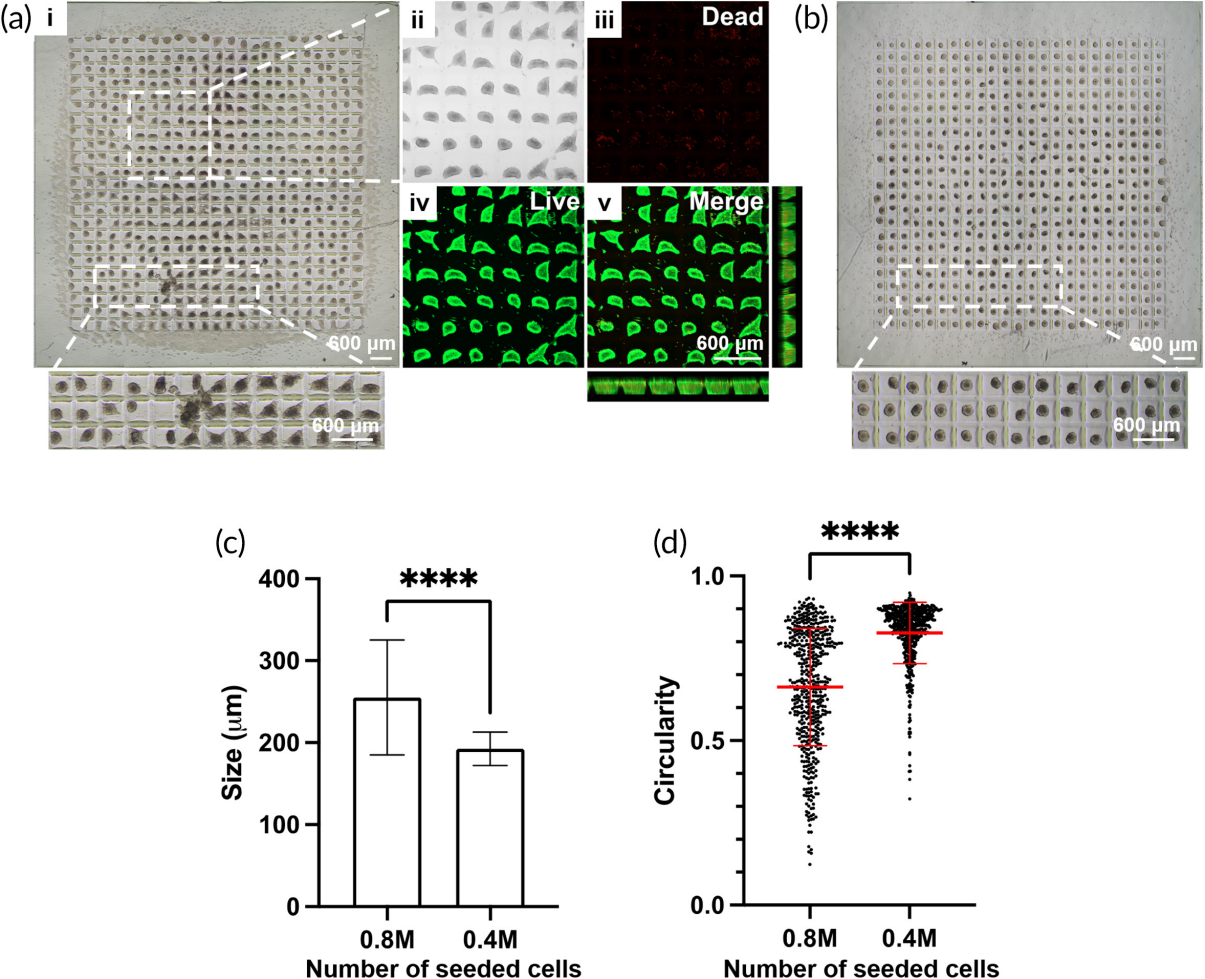
In situ formation of microtissues on S‐300 GelMA “lock” component. Bright field and confocal images after live/dead fluorescent staining of INS‐1E microtissues formed on S‐300 GelMA “lock” component from (a) 0.8 million of seeded cells and (b) 0.4 million of seeded cells. Images were acquired 24 h following cell seeding. Scale bar representing 600 μm in image a (v) applies to a (ii–iv). Quantification of (c) size and (d) circularity for INS‐1E microtissues formed by in situ assembly of monodispersed INS‐1E cells on S‐300 GelMA “lock” component. (****) denotes statistical difference (*p* < 0.0001).

Figure 6a showed the image of an S‐300 GelMA “lock” component on which 0.8 million of monodispersed INS‐1E cells suspended in culture medium were dispensed. Most cells were distributed into the microwells within the GelMA “lock” component and eventually sank before clustering (Figure 6A(ii)). After 24 h of incubation, most microwells contained one assembled microtissues each while the cells that resided near the external edges outside of the micropatterned GelMA “lock” did not aggregate (Figure 6a(i)). Confocal microscopy after fluorescent live/dead staining of the microtissues confirmed that they were highly viable and located completely within the interior of the microwells in a uniform array, as shown by their projected confocal images (Figure 6a(iii–v)). However, the microtissues were irregularly shaped and each exhibited a pointed protrusion towards the corners of the microwells. We postulate that this cell assembly behavior might be due to the increased attachment of cells at the peripheral of microtissues to the stiffer substrate^43^ associated with exposure to a higher UV intensity^44^ at the microwell corners. The in situ microtissue formation was further optimized by reducing the number of cells dispensed into each S‐300 GelMA “lock” component to 0.4 million cells per device. With this lower number of seeded cells, the microtissues formed after 24 h exhibited a more spherical shape and remained free of pointed protrusion or attachment to microwell corners (Figure 6b). Specifically, microtissues assembled in situ from 0.4 million cells exhibited a higher average circularity of 0.83 (Figure 6d) with less variation in size (Figure 6c) compared to those assembled from 0.8 million cells with an average circularity of 0.66. This altered behavior in cell assembly might be due to the presence of a smaller number of mono‐dispersed cells per microwell, facilitating cellular clustering at the center of the microwell to form uniform, circular microtissues while minimizing attachment at the microwell corners.

Overall, the data in Figure 6 demonstrated that the micropatterned waffle‐inspired design of the GelMA “lock” facilitated in situ microtissue formation from monodispersed cells. This in situ microtissue assembly approach enabled fabrication of a device with maximal microtissue entrapment efficiency of nearly 100%. This in situ strategy also optimized spatial distribution to maximize the likelihood that each microwell would be occupied by a single microtissue to achieve a microtissue distribution ratio of about 100%. This scenario is ideal to prevent microtissue aggregation and potentially enhance nutrient access for the embedded microtissues.

### 3.7 Incorporation of spatially guided vascular‐inductive cells into GelMA “lock” component

**FIGURE 7:**
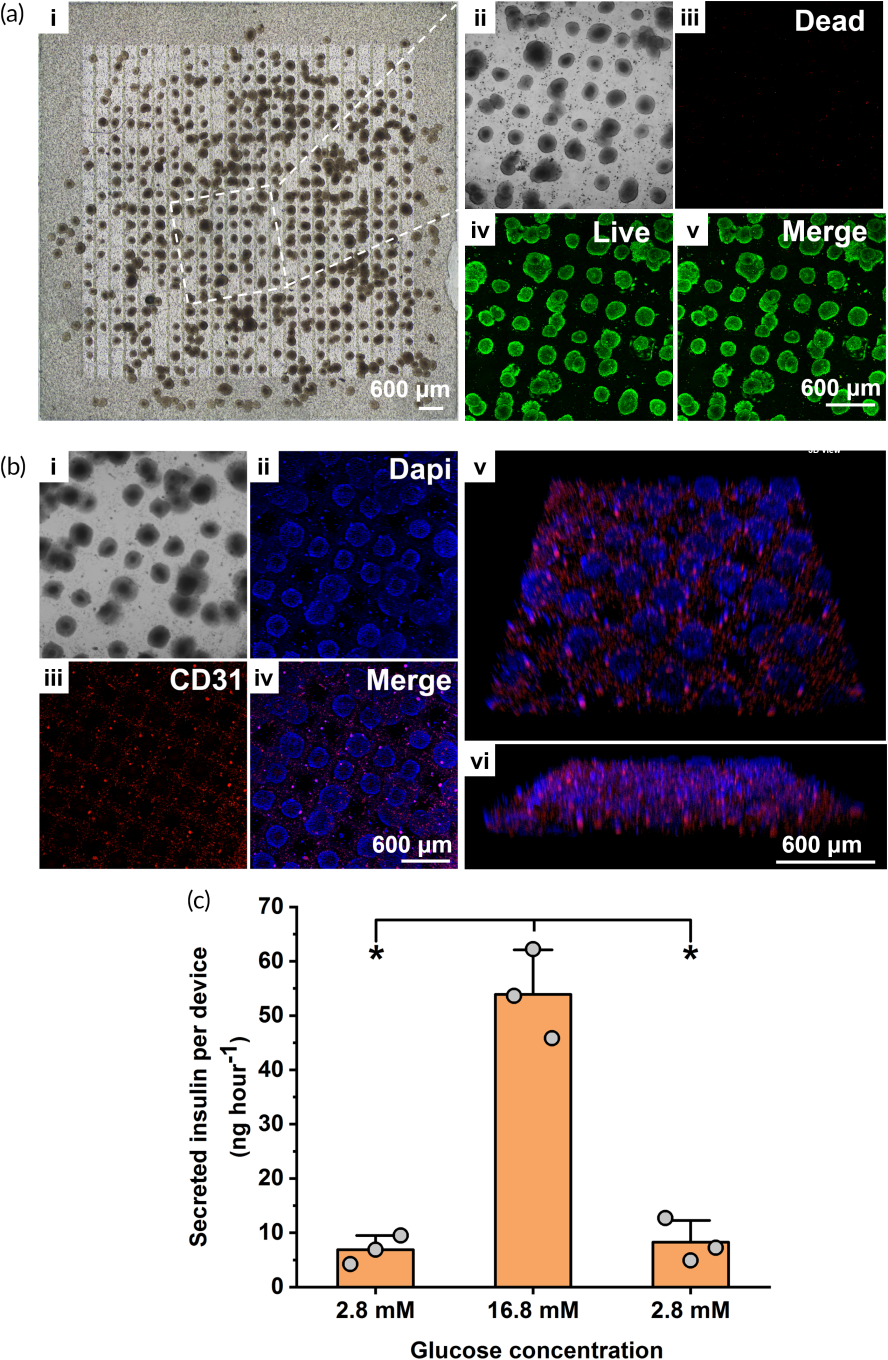
In vitro viability and function of INS‐1E microtissues and HUVECs co‐laden in S‐300 WIM device (a) Bright‐field image (i) and projections of three‐dimensional (3D) confocal image stacks (ii–v) of HUVEC/INS‐1 E microtissues co‐laden in S‐300 WIM device after fluorescent live/dead staining. Images were acquired 18 h after device fabrication. Scale bar representing 600 μm in image a (v) applies to a (ii–iv). (b) Projections of 3D confocal image stacks (i–iv) and snapshot of 3D view of S‐300 WIM device after CD31 immunofluorescent staining (v‐vi). Scale bar representing 600 μm in image b (iv) applies to b (i–iii). Scale bar representing 600 μm in image b (vi) applies to image b (v). (c) Static glucose stimulated insulin secretion of microtissues during consecutively incubations in different glucose concentration for 1 h at each concentration. Data are shown as mean ± SD (*n* = 3 replicate samples). Data are representative of *N* = 2 independent experiments. (*) denotes statistical difference (*p* < 0.05).

The WIM device could be further enhanced by incorporating neovascularization which might facilitate passive transportation of nutrients and oxygen to the encapsulated microtissues. Specifically, we postulate that the micropatterned waffle‐inspired “lock” of GelMA, which is a substrate suitable for cellular encapsulation and proliferation,^45^ could be loaded with vascular‐inductive cells such as HUVECs to support their development into new vasculature. Therefore, we examined the feasibility of embedding primary HUVECs in the GelMA sidewalls of the S‐300 “lock” component prior to encapsulating therapeutic microtissues and evaluated the viability of these loaded HUVECs using fluorescent live/dead staining assay followed by confocal microscopy **(**Figure S12a). HUVECs were mixed with the GelMA prepolymer solution prior to the fabrication of the S‐300 “lock” component (Figure S1) following in vitro culture for 7 days, embedded HUVECs exhibited good cellular viability and pro‐angiogenic function as indicated by CD31 expression within the GelMA “lock” component (Figure S12b). This observation suggested that the waffle‐inspired interconnected micropattern of the GelMA “lock” component has the potential to provide controlled and uniform spatial guidance for the subsequent growth of an organized vascularized system throughout the entire device to ultimately reach individual therapeutic microtissues entrapped in the microwells. Specifically, HUVECs embedded in S‐300 “lock” components (Figure S13) fabricated using 5% and 7.5% (w/v) GelMA formed vessel‐like structures after 2 days of in vitro culture while endothelial sprouting was also observed for HUVECs embedded in S‐300 “lock” component fabricated from 10% (w/v) GelMA. However, this strategy of co‐loading an additional vascular inductive cell type adjacent to the therapeutic cell type within the same encapsulation device also raises arguable concern for potential competition for oxygen or adverse cellular interaction between these two cell types. Therefore, we subsequently investigated the feasibility of co‐loading both vascular‐inductive HUVECs and insulin‐secreting INS‐1E microtissues in the same S‐300 WIM device by evaluating cellular viability and preservation of intended function for each cell type to address such concern (Figure 7).

#### 3.7.1 Viability assessment of co‐laden therapeutic and vascular‐inductive cells

An S‐300 WIM device co‐encapsulating both cell types was fabricated by deposition of a mixture of alginate and INS‐1E microtissues on the HUVEC‐embedded S‐300 “lock” component that has been maintained at standard tissue culture conditions for 7 days (Figure 7a). The viability of HUVECs and INS‐1E microtissues co‐laden in the S‐300 WIM device (Figure 7a(i)) were determined by live/dead fluorescent assay after overnight incubation following device fabrication (Figure 7a(ii–v)). Confocal imaging showed highly viable INS‐1E microtissues visualized predominantly with green fluorescence (Figure 7a(iv)) and only a negligible number of dead cells with red signal (Figure 7a(iii)). Interestingly, HUVECs embedded in the GelMA sidewalls could not be clearly visualized in Figure 7a(iv), probably due to the much weaker fluorescent signals from the less densely packed HUVECs adjacent to compact INS‐1E microtissues, rendering it difficult to capture signals from both cell types at the same confocal laser intensity. Nonetheless, HUVECs residing near the outermost GelMA edge of the device far away from neighboring INS‐1E microtissues could be visualized more clearly (Figure **S14**) as green fluorescent dots of viable cells while a negligible red fluorescent signal within the same field of view indicated negligible HUVEC death.

#### 3.7.2 Evaluating the function of co‐laden vascular‐inductive cells in expressing pro‐angiogenetic marker

The effect of co‐loading both cell types within the same S‐300 WIM device on cellular functions, specifically the vascular‐inductive potential of HUVECs (Figure 7b) and insulin secretion function of INS‐1E microtissues (Figure 7c), were further investigated. Pro‐angiogenic marker CD31, which is also known as platelet endothelial cell adhesion molecule 1 (PECAM‐1), was first identified as a surface marker on endothelial cells and was enriched at intercellular junctions.^46^, ^47^ CD31 was frequently used as a marker for endothelial presence and function due to its role in the survival, migration, and barrier maintenance of endothelial cells despite its concurrent expression in other cell types.^47^ Since HUVECs were the only cell type expressing CD31 marker in the WIM device, any positive detection of CD31 would demonstrate the preserved pro‐angiogenic potential of the co‐laden HUVECs. Red immunofluorescent staining for CD31 was performed for WIM device (Figure 7b(iii)) co‐encapsulating both cell types while cellular nuclei in the same device were stained with DAPI (Figure 7b(ii)). Red fluorescence (Figure 7b(iii)) observed throughout the entire GelMA “lock”component indicated the extensive presence of CD31^+^ angiogenetic markers in spatially guided distribution along the micropatterned waffle‐inspired network of the GelMA “lock” component. Co‐localization of red fluorescence from CD31 with blue fluorescence from DAPI‐stained nuclei resulted in magenta signal in the merged image (Figure 7b(iv)), demonstrating the presence of CD31^+^ HUVECs and thus confirming that the embedded HUVECs retained their pro‐angiogenic potential. Figure 7b showed snapshots of 3D view of the co‐laden S‐300 device at different angles. Figure 7b(v) showed that CD31^+^ HUVECs visualized with magenta fluorescent signals evenly interspersed with blue‐fluorescent microtissues throughout the device. Figure 7b(vi) also showed the positions of vascular‐inductive cells and microtissues in a co‐planar arrangement, confirming that the interlocking of the GelMA “lock” and alginate “key” components facilitated placement of these two cell types in desired proximity. Overall, the S‐300 WIM device design preserved the vascular‐inductive potential of HUVECs while placing them in proximity to INS‐1E microtissues.

#### 3.7.3 Evaluating glucose‐stimulated insulin secretion of co‐laden therapeutic microtissues

Functional performance of INS‐1E microtissues co‐laden with HUVECs in the S‐300 WIM device was also characterized with GSIS assay (Figure **7**c **)**. The encapsulated microtissues demonstrated a 8.5‐fold increase in the amount of secreted insulin after exposure to the high glucose level of 16.8 mM following a prior incubation in 2.8 mM glucose solution (Figure S8). After the third incubation at the low glucose level, the device also showed an adjustment of insulin secretion back to the basal level. This response was similar to the pattern of insulin secretion of S‐300 WIM device without embedded HUVECs (Figure 4b), confirming that the combination of HUVEC and INS‐1E in the WIM device did not cause undesirable effects on the function of insulin‐secreting microtissues.

## 4 DISCUSSION

Overall, we herein designed and fabricated an encapsulation device enabling homogeneous spatial distribution of therapeutic microtissues and a waffle‐inspired GelMA “lock” component supporting pro‐angiogenic endothelial cells towards the future goal of facilitating transfer of oxygen and nutrients to the encapsulated microtissues. Uncontrolled spatial distribution of multicellular microtissues in close proximity can induce microtissue aggregation and fusion thus leading to formation of larger tissue structures. This fusion increases diffusion distance, resulting in diminished nutrients and oxygen transport to the innermost cells and ultimately decreases their viability. ^9c,18b^ Homogenously distributing one microtissue in each microwell of the WIM device will prevent direct contact between adjacent microtissues and thus is likely to reduce the risk for microtissue aggregation. Among the three WIM devices evaluated (Figure 2d), S‐300 WIM device presented a higher microtissue distribution ratio for microwells of type M_1_ compared to the other two devices, indicating that the S‐300 GelMA “lock” component was the optimal design to maximize homogeneous spatial distribution of microtissues. S‐300 WIM device could accommodate a higher number of microwells per unit device area due to the smaller size of its individual microwell in comparison to that of S‐400 or C‐400 devices. Furthermore, the individual microwell of the S‐300 WIM device, albeit with a microwell side length of 300 μm being the smallest among that of all three devices, was still sufficiently large to adequately accommodate INS‐1E microtissues with an average diameter of 150 μm. This combination of sufficient microwell dimension and a higher number of microwells available per unit area thus synergistically facilitated optimal spatial distribution of microtissues to maximize the probability of one microtissue being entrapped in one microwell. Furthermore, 2 days following device fabrication (Figure S7), S‐300 WIM device maintained homogeneous spatial distribution of microtissues by spatially “locking” them in microwells with no observed microtissue fusion. In contrast, control devices with microtissues embedded in a monolithic alginate block wrinkled and shrank immediately after device fabrication, causing microtissues clustering and aggregation. Therefore, the S‐300 WIM device was selected for further evaluation in subsequent experiments of this study.

We further demonstrated that transplanting the alginate‐coated S300 WIM devices containing primary rat islets at a dose of 500 IEQs per mouse resulted in glycemic correction in chemically induced, immuno‐competent diabetic mice for up to 2 weeks. This data (Figure 5) was sufficient to support our conservative claim that the dimension of the alginate‐coated S‐300 WIM device allowed sufficient release of insulin in vivo to restore blood glucose control with a relatively low dose of rat islets. Arguably, though the duration of glycemic correction in for the in vivo study might be considered relatively short, this data were sufficient to demonstrate that the alginate‐coated WIM device has adequately shielded the transplanted rat islets from destruction by the immune system of diabetic mice during this period of time. Specifically, if the rat islets were not sufficiently protected from the immune system of immunocompetent diabetic mice, islet death would have occured within the first few days resulting in immediate loss of blood glucose control as observed in published studies.^48^, ^49^ Furthermore, it is important to highlight that the duration of glycemic correction depends on multiple factors. Specifically, comparing duration of glycemic control for different device designs requires standardization of therapeutic dosage, the partial oxygen pressure at the implant site and the presence of any enhancing factor such as oxygen‐generating materials or supporting cells. Published studies using a higher dose of rat islets or alternative implant sites with higher partial oxygen pressure P_O2_ such as the intraperitoneal space have resulted in treatment duration of months or longer.^35^ However, most reported macrodevices that were implanted at the oxygen‐poor subcutaneous site with a therapeutic dosage 500 IEQs of primary rat islets per immuno‐competent mouse but without any enhancing factors either failed^50^ or only achieved glycemic correction for up to 10 days compared to our reported 2‐week duration.^51^ Nonetheless, we acknowledge that the spatial distribution of primary rat islets within the transplanted device might not be ideally homogeneous due to the geometrical variation in shapes and sizes of these native microtissues (Figures 5b–d, S9). Specifically, the low circularity of primary rat islets (0.76) compared to INS‐1E microtissues fabricated ex situ (0.85) (Figure S9) which resulted in poorer spatial distribution observed in the WIM device (Figure 5d). Furthermore, large islets are inherently susceptible to hypoxia and necrotic cores,^42^ diminishing their survival in vivo and thus their long‐term efficacy in diabetes correction.

To address the challenge of nonuniform geometry of therapeutic microtissues, multiple prior studies have previously demonstrated the feasibility of reaggregating monodispersed cells derived from large primary islets or stem cell‐derived beta cell clusters^42^, ^52^, ^53^ into smaller and more uniform microtissues with improved viability.^42^, ^53^ In this study, we demonstrated that “on device” assembly of monodispersed INS‐1E cells on S‐300 GelMA “lock” component **(**Figure 6 **)** resulted in in situ formation of microtissues with more uniform size and improved circularity **(**Figure 6 **)** compared to primary rat islets, thus facilitating more homogenous spatial distribution. Thus, the WIM device as an in situ platform, which enables both reaggregation of mono‐dispersed insulin‐secreting cells into uniform microtissues and their encapsulation in semi‐permeable alginate (Figure 6 **)**, has the potential to achieve both superior cellular viability and optimal microtissue distribution homogeneity. This proof‐of‐concept demonstration with microtissues assembled from INS‐1E cells **(**Figure 6 **)** lays a foundation for further studies of a next‐generation macrodevice. We envision a future study to evaluate long‐term in vivo efficacy for an improved device containing therapeutic microtissues, which are assembled in situ from a more clinically relevant cell source such as as beta‐like clusters derived from human‐induced pluripotent stem cells. We postulate that spatially homogeneous distribution of such therapeutic microtissues will result in differential cellular viability and functionality during assessment of long‐term in vivo efficacy. Additional monitoring of in vivo insulin plasma level and ex vivo characterization of the retrieved macrodevice including histological analysis for immuno‐compatibility and examination of microtissue morphology will also provide more comprehensive understanding of the device performance in preserving microtissue viability and performance.

In addition, prior publications have demonstrated that synthetic polymers such as poly (ethersulfone) (PES)/polyvinyl pyrrolidone (PVP) blend can be utilized to fabricate different microwell designs to facilitate spatial distribution of microtissues.^8^, ^16^ However, such strategy does not support the growth of an embedded vasculature network as pro‐angiogenic endothelial cells cannot be incorporated into the dense walls of these hydrophobic polymers. Since GelMA was shown to support survival and function of endothelial cells,^32^, ^33^ we anticipated that the HUVEC‐embedded GelMA matrix of the S‐300 WIM device would mature into a penetrating vascular network co‐planar with the encapsulated microtissues in future in vivo study. Notably, other strategies for device vascularization have not achieved this co‐planar spatial arrangement of both pro‐angiogenic and therapeutic cell types,^54^ theoretically rendering it more difficult for oxygen and nutrients to diffuse to microtissues located farther away from the vascular network. This study demonstrated that the WIM device design enabled preservation of the vascular‐inductive potential of HUVECs while placing them in proximity to INS‐1E microtissues (Figure 7). Expression of pro‐angiogenic marker CD31 by HUVECs co‐laden with insulin‐secreting microtissues in a co‐planar arrangement within the S‐300 WIM device suggested the possibility of forming capillary‐like structures by embedding vascular‐inductive endothelial cells in the GelMA network. After 2 days of in vitro culture (Figure S13), even though a more mature capillary‐like network could be formed from HUVECs embedded in S‐300 GelMA “lock” components that were fabricated from lower concentrations of 5% and 7.5% (w/v) of hydrogel prepolymers, endothelial sprouting was also observed when a higher concentration of 10% (w/v) was used. This observation aligned with published studies demonstrating that sprouting of embedded HUVECs into 3D capillary network is facilitated by decreased stiffness of GelMA hydrogels formed from lower prepolymer concentrations.^55^ In this study, the S‐300 GelMA “lock” component was fabricated with the higher prepolymer concentration of 10% (w/v) to provide sufficient stiffness and thus mechanical strength for device handling. Nonetheless, we speculate that, upon device transplantation, in vivo degradation of GelMA might eventually decrease its stiffness to support maturation of endothelial sprouting into formation of capillary network.^54^ Previous studies demonstrated that crosslinked GelMA hydrogel provided favorable environment for cellular proliferation and spreading due to its RGD motifs which were responsive to matrix metalloproteinases and supportive of cell attachment.^45^ Nikkhah *et al*. also reported vascularization of linear GelMA microstructures with embedded HUVECs.^56^ Furthermore, coculture of human vascular cells and human mesenchymal stem cells in GelMA hydrogel generated lumen‐containing vascular network in vitro.^57^ After its implantation into immunodeficient mice, this cell‐laden GelMA hydrogel construct resulted in anastomosis between mouse vasculature and in vitro cultured human vascular network.^57^ Together, these literature evidences corroborate the potential application of our WIM device design as a promising strategy to create an interconnected network of neo‐vasculature interspersing therapeutic microtissues to enhance their performance.

The mechanical integrity of the WIM device is another aspect requiring further investigation. In this study, the S‐300 WIM device without an additional alginate coating was sufficiently robust to be handled by a pair of tweezers for static in vitro evaluation with confocal microscopy or GSIS assay. Incorporation of the GelMA “lock” component with waffle‐inspired micropattern improved the structural integrity of the uncoated WIM device (Figure 3bi) compared to a control device (Figure 3bii), which comprised microtissues encapsulated in a monolithic alginate block without the GelMA “lock.” As shown in (Figure 3bii), such control device wrinkled and did not maintain its intended square shape due to the absence of a GelMA network, which could have otherwise acted as a backbone framework to prevent the hydrogel from wrinkling. Nonetheless, in a pilot in vitro experiment during which the uncoated S‐300 WIM device was subjected to shaking motion to mimic potential mechanical challenge of the in vivo condition such as animal scratching behavior or body movement, we observed detachment of the two device components. Therefore, we designed a modified protocol to add an additional alginate coating to mechanically strengthen the device and preserve its structural integrity upon in vivo transplantation. For future development of next‐generation devices, we postulate that the mechanical strength of the WIM device might be improved by incorporation of mechanically robust materials into the “lock” component of this design. For example, electrospun nanofibers might be incorporated to the GelMA “lock” component by mixing these fibers with GelMA prepolymers prior to UV crosslinking to enhance the strength of the micropatterned hydrogel. Alternatively, free‐standing nanofibrous electrospun membrane formed by conformal deposition on an alloy template can also be employed as a robust structural framework to support the GelMA “lock” component.^58^ Furthermore, additive manufacturing of more mechanically robust polymers by melt electrowriting or extrusion printing might also be utilized to print the waffle‐inspired design of the “lock” component.^59^, ^60^ Nonetheless, it would generally be more challenging to achieve the same resolution of the sidewall dimension by these 3D printing techniques instead of the lithography‐based patterning of GelMA as demonstrated in this study.

Lastly, delivery of a clinically relevant dosage of therapeutic cells while maintaining the spatially homogeneous distribution of the encapsulated microtissues remain an unsolved challenge for our current WIM device. Nonetheless, this scalability issue is currently a universal challenge for most reported macro‐encapsulation systems which also require large device size for translation of preclinical success into clinically relevant applications.^61^ This study focused only on demonstrating the proof‐of‐concept that a waffle‐inspired “lock” component could distribute microtissues evenly in a 2D arrangement. Similar approach might also be adopted to design a next‐generation device to homogeneously distribute the microtissues in a 3D arrangement by stacking multiple layers each comprising co‐planar therapeutic microtissues and endothelial cells. Such design might increase microtissue packing density thus reducing device area required while minimizing microtissue fusion for improved cellular viability.

## 5 CONCLUSION

We fabricated a macrodevice to position encapsulated therapeutic microtissues in spatially homogeneous distribution while preserving their cellular viability and insulin‐secretion function. The waffle‐inspired micropattern of the GelMA“lock” component in the WIM device directed distribution of microtissues by effectively entrapping them in microwells to mitigate microtissue reaggregation. Transplantation of the alginate‐coated S‐300 WIM device encapsulating primary rat islets resulted in glycemic correction in diabetic immunocompetent mice for 2 weeks. The GelMA “lock” component also guided assembly of mono‐dispersed cells to form uniform microtissues in situ with maximal entrapment efficiency. Furthermore, vascular inductive cells could be incorporated in the sidewalls of the GelMA lock‐component in a co‐planar arrangement with close proximity to therapeutic microtissues. These two co‐laden cell types maintained their viability and function. Specifically, the WIM device co‐laden with INS‐1E microtissues and HUVECs maintained desirable cellular viability in vitro with encapsulated microtissues retaining their glucose‐responsive insulin secretion while embedded HUVECs expressed pro‐angiogenic markers. Together, this study motivates future long‐term evaluation of a next generation WIM device as a platform for cellular delivery in preclinical animal models to further assess its potential therapeutic efficacy for the treatment of diabetes or other protein‐deficiency diseases.

## AUTHOR CONTRIBUTIONS

**Chi H. L. Pham:** Formal analysis (equal); investigation (lead); methodology (lead); resources (equal); validation (equal); visualization (equal); writing – original draft (equal); writing – review and editing (equal). **Yicong Zuo:** Conceptualization (lead); formal analysis (lead); investigation (lead); methodology (lead); resources (lead); validation (lead); writing – original draft (lead). **Yang Chen:** Investigation (equal); resources (equal); visualization (equal); writing – review and editing (equal). **Nam M. Tran:** Resources (supporting); writing – review and editing (equal). **Dang T. Nguyen:** Investigation (equal); methodology (equal); resources (supporting). **Tram T. Dang:** Conceptualization (lead); funding acquisition (lead); project administration (lead); supervision (lead); writing – review and editing (lead).

## CONFLICT OF INTEREST STATEMENT

The authors have no conflicts of interest to declare.

### PEER REVIEW

The peer review history for this article is available at https://publons.com/publon/10.1002/btm2.10495.

## ETHICS STATEMENT

This study is approved by the Institutional Animal Care and Use Committee (IACUC) of Nanyang Technological University (NTU), Singapore (A19023) on May 2019. All animal experiments followed the National Advisory Committee for the Laboratory Animal Research (NACLAR), which complies with the National Institutes of Health guide for the care and use of laboratory animals (NIH Publications No. 8023, revised in 1978).

## Supporting information


**Appendix S1:** Supporting information

## Data Availability

Data available on request from the authors
