# Supplementary material for: Waffle‐inspired hydrogel‐based macrodevice for spatially controlled distribution of encapsulated therapeutic microtissues and pro‐angiogenic endothelial cells
Source: Bioeng Transl Med. 2023 Mar 14;8(3):e10495. doi: 10.1002/btm2.10495 (PMC10189477; doi:10.1002/btm2.10495)
Supplement: Supplementary file 1 — Appendix S1: Supporting information [file BTM2-8-e10495-s001.docx]

**APPENDIX**

**Supporting Information**

# **Waffle-inspired hydrogel-based macrodevice for spatially controlled distribution of encapsulated therapeutic microtissues and pro-angiogenic endothelial cells**

*Chi H.L. Pham^+^, Yicong Zuo^+^, Yang Chen^+^, Nam M. Tran, Dang T. Nguyen, Tram T. Dang**

School of Chemical and Biomedical Engineering, Nanyang Technological University (NTU), Singapore 637459, Singapore

*(+) denotes equal contribution*

*(*) denotes corresponding author*

Email: ttdang@ntu.edu.sg

### I. Supplementary method

***Quantitative analysis of distribution of microtissues encapsulated in WIM*** *device:* Three parameters were evaluated by quantitatively analyzing the bright-filed images of the microtissue-encapsulating WIM devices using ImageJ.

**Microtissue entrapment efficiency** = $\frac{N_{in}}{N_{microtissue}}$ × 100% (1)

where *N_in_* is the total number of microtissues effectively entrapped within the interior of the microwells as illustrated in **Figure S5** and *N_microtissue_* is the total number of microtissues encapsulated in the entire WIM device including those on the sidewalls and outer edges of the WIM device.

**Unoccupied microwell ratio** = $\frac{N_{unoccupied}}{N_{microwell}}$ × 100% (2)

where *N_unoccupied_* is the total number of unoccupied microwells per device and *N_microwell_* is the total number of microwells per device.

**Microtissue distribution ratio for microwells of type M_n_** = $\frac{\text{(n × W}\text{n}\text{)}}{N_{microtissue}}$ × 100% (3)

where M_n­_ is the type of microwells containing *n* microtissues per well with *n* ranging from 1 to 6 and W_n_ is the total number of the microwells of type M_n_.

### II. Supplementary figures

***Figure S1****. Schematics of the procedure for patterning of the GelMA “lock” component using photolithography*

**
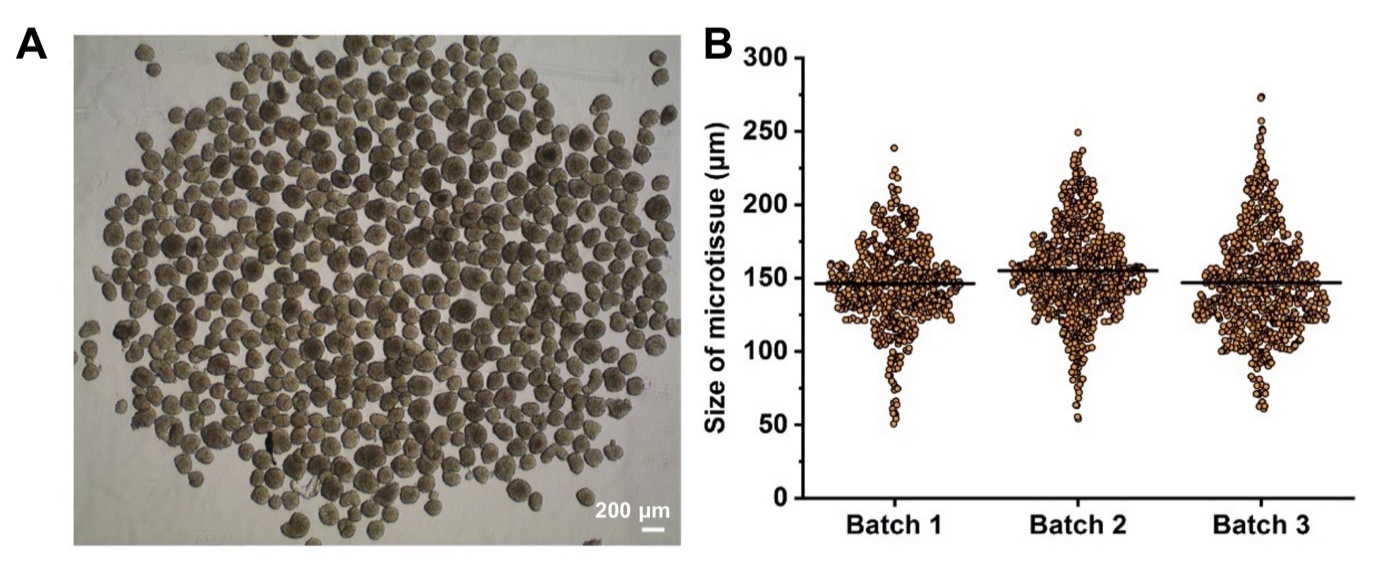
**

***Figure S2****. Size homogeneity of encapsulated spheroidal INS-1E microtissues.* ***(A)*** *Bright-field images of spheroidal INS-1E microtissues fabricated ex situ using agarose micromolds.^32^ Image was acquired immediately after microtissue retrieval from the agarose micromolds.* ***(B)*** *Size distribution of spheroid INS-1E microtissues. Scale bar: 200 μm.*

*
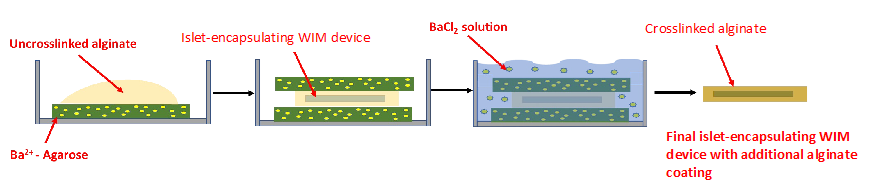
*

***Figure S3****. Schematic illustration of the additional alginate coating of an islet-loaded WIM device. The islet-loaded WIM device in step (b) was obtained upon completion of the fabrication procedure depicted in Figure 1. The Ba^2+^-enriched agarose disks provided the weight that flattened the layer of the alginate solution surrounding the WIM-device to mitigate the effect of surface tension which would otherwise result in a curved coating layer with increased thickness at the center of the final device. The Ba^2+^-enriched agarose disks also provided a supply of Ba^2+^ ions that diffused uniformly throughout the entire alginate coating.*


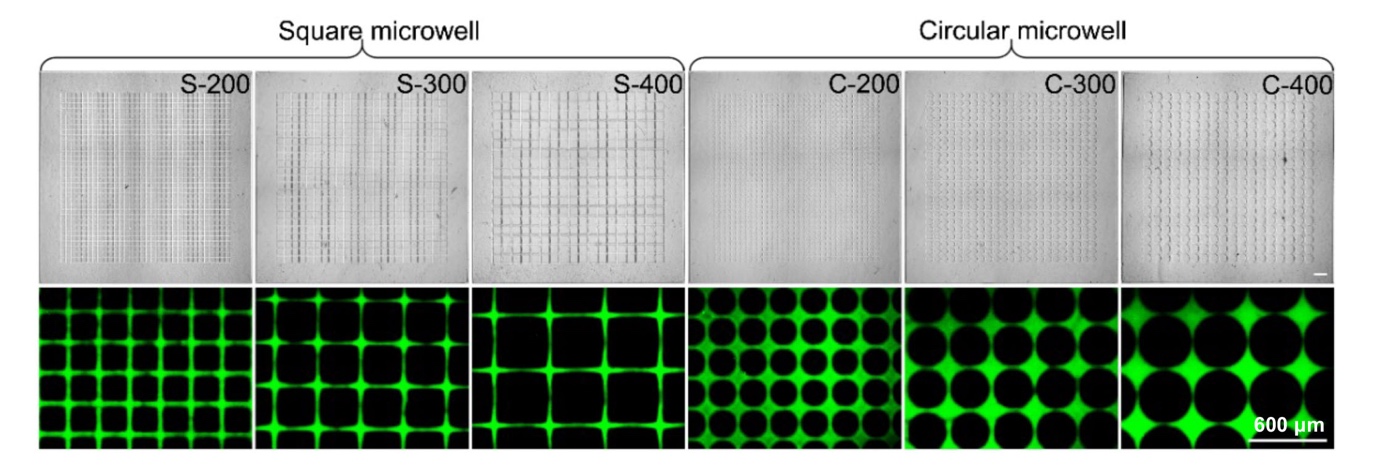


***Figure S4.*** *Optimizing design parameters of micropatterned GelMA “lock” component with varying microwell geometries and dimensions. Arrays of both square microwells each with a sidelength varying from 200-400 µm or circular microwells each with a diameter varying from 200-400 µm were fabricated from GelMA hydrogel. Top and bottom panels showed the bright-field and fluorescent images of corresponding GelMA patterns respectively. Scale bar: 600 μm.*


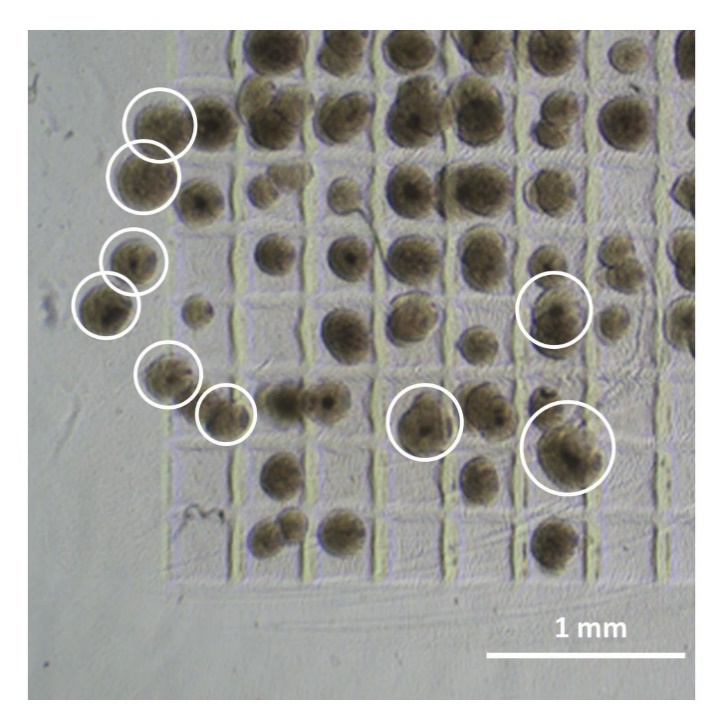


***Figure S5.*** *Magnified optical image of a region of interest on a S-300 WIM device illustrating criteria used to determine effective entrapment of microtissues within microwells for calculation of microtissue entrapment efficiency.* *Untrapped microtissues settling on sidewalls or outer edge of S-300 WIM device are indicated with white circles. Remaining uncircled microtissues with at least 50% of their volume confined within microwells were deemed effectively entrapped in the interior of the microwells. Scale bar: 1mm.*


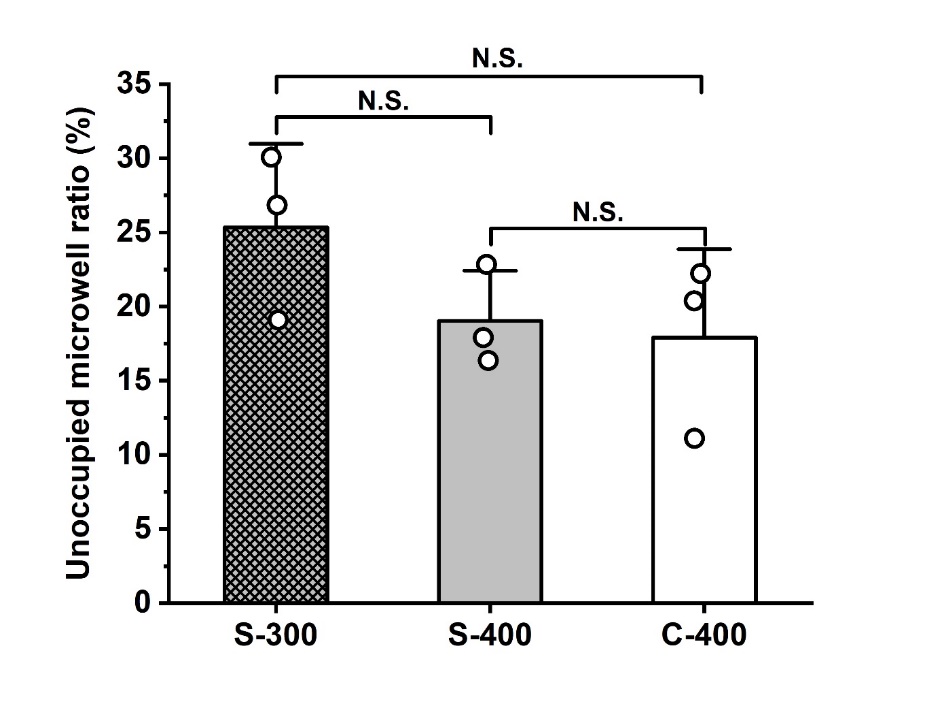


***Figure S6.*** *Effect of design parameters for GelMA “lock” component on unoccupied microwell ratio. This ratio is defined as the ratio of the number of empty microwells to the total number of microwells in each “lock” component. No statistical significance was observed for unoccupied microwell ratios from all GelMA “lock” components of S-300, S-400 and C-400.*

**

***Figure S7****. Optical images of S-300 WIM device and control device, both encapsulating INS-1E microtissues after 2 days of in vitro culture following device fabrication. Data is representative for n = 3 devices. S-300 WIM device maintained homogeneous spatial distribution of microtissues by spatially “locking” them in microwells with no observed microtissue fusion after 2 days. In contrast, control devices with microtissues embedded in a monolithic alginate wrinkled and shrank immediately after device fabrication, causing microtissues clustering and aggregation.*

*
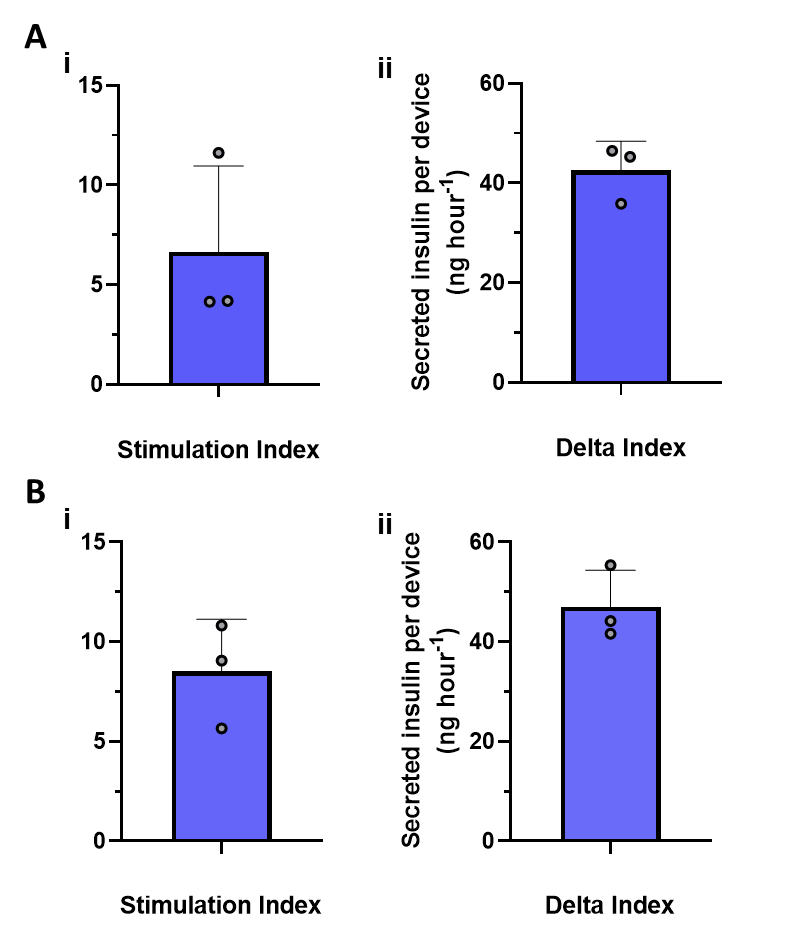
*

***Figure S8****. Stimulation and Delta indices of INS-1E microtissues encapsulated in S-300 WIM device demonstrated glucose-responsive insulin secretion. Figure S8.A (i-ii) was derived from the same GSIS data presented in Figure 4B while Figure S8.B(i-ii) was similarly derived from Figure 7B. Data is shown as mean ± SD (n=3 replicate samples). Data is representative of N=2 independent experiments.*

*
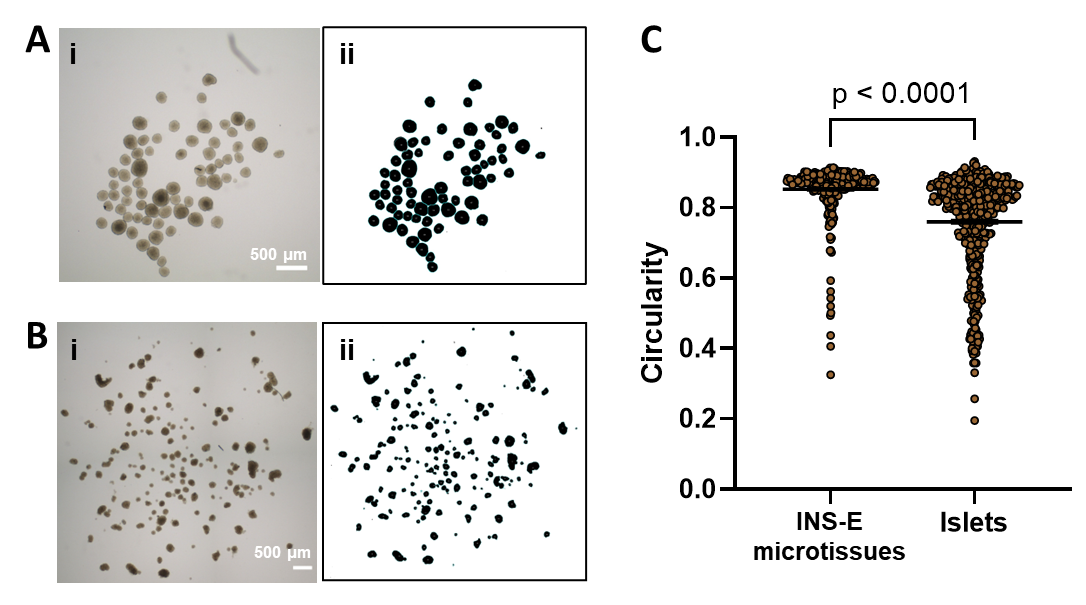
*

***Figure S9.*** *Circularity measurement of* *INS-1E microtissues and primary rat islets. (****A, B****) Bright-field (i) and measurement record image (ii) showing islet geometry automatically detected with particle analysis function from ImageJ software of INS-1E microtissues (A) and rat islets(B), respectively. (****C****) Circularity distribution of INS-1E microtissues (n=313) and rat islets (n=496). Scale bars: 500 μm.*

*
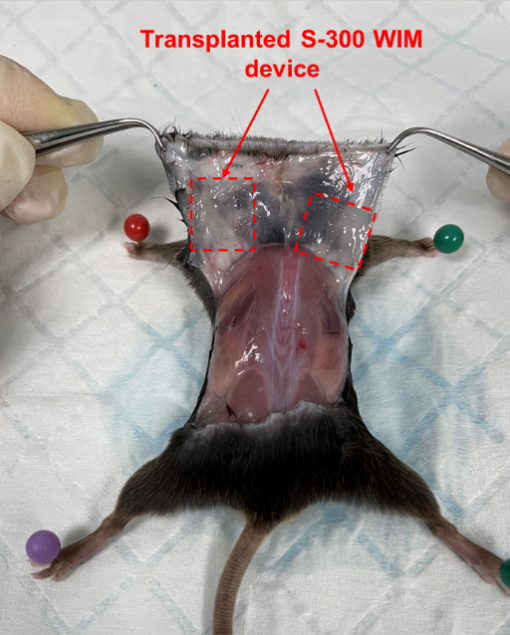
*

***Figure S10****. Blank alginate-coated S-300 WIM devices without islets maintained structural integrity at 2 weeks following subcutaneous transplantation in an immunocompetent chemically induced diabetic C57/B6J mouse.*

**
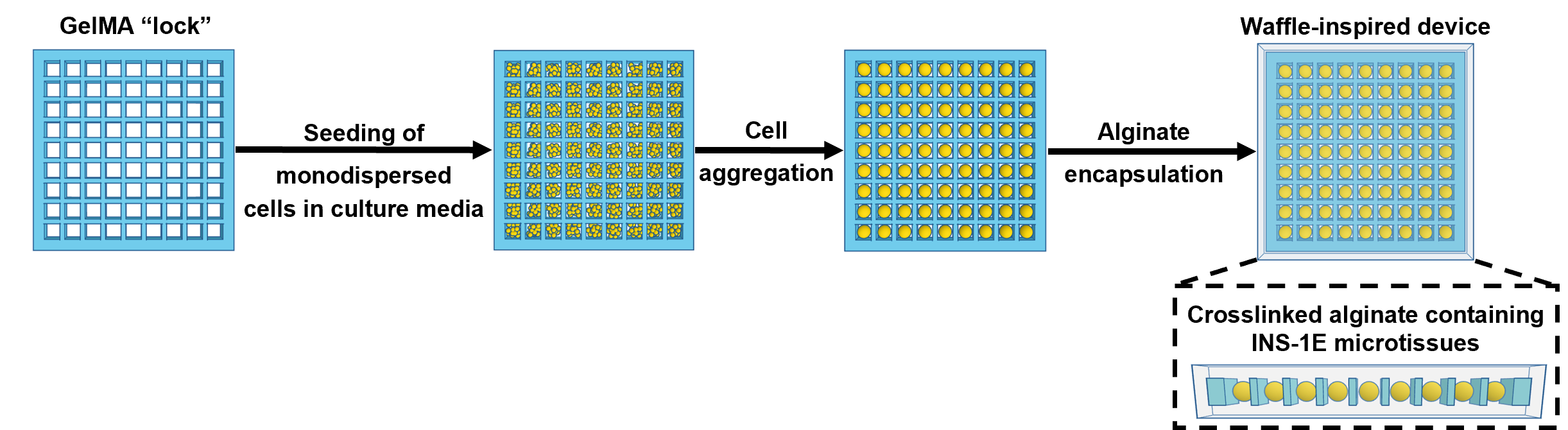
**

***Figure S11.*** *Schematics illustrating procedure for in situ fabrication of the WIM device. “On device” assembly of microtissues from mono-dispersed cells due to spatial guidance by the waffle-inspired micropattern of the GelMA lock component and subsequently encapsulation of the formed microtissues in alginate hydrogel to form the final WIM device.*

*
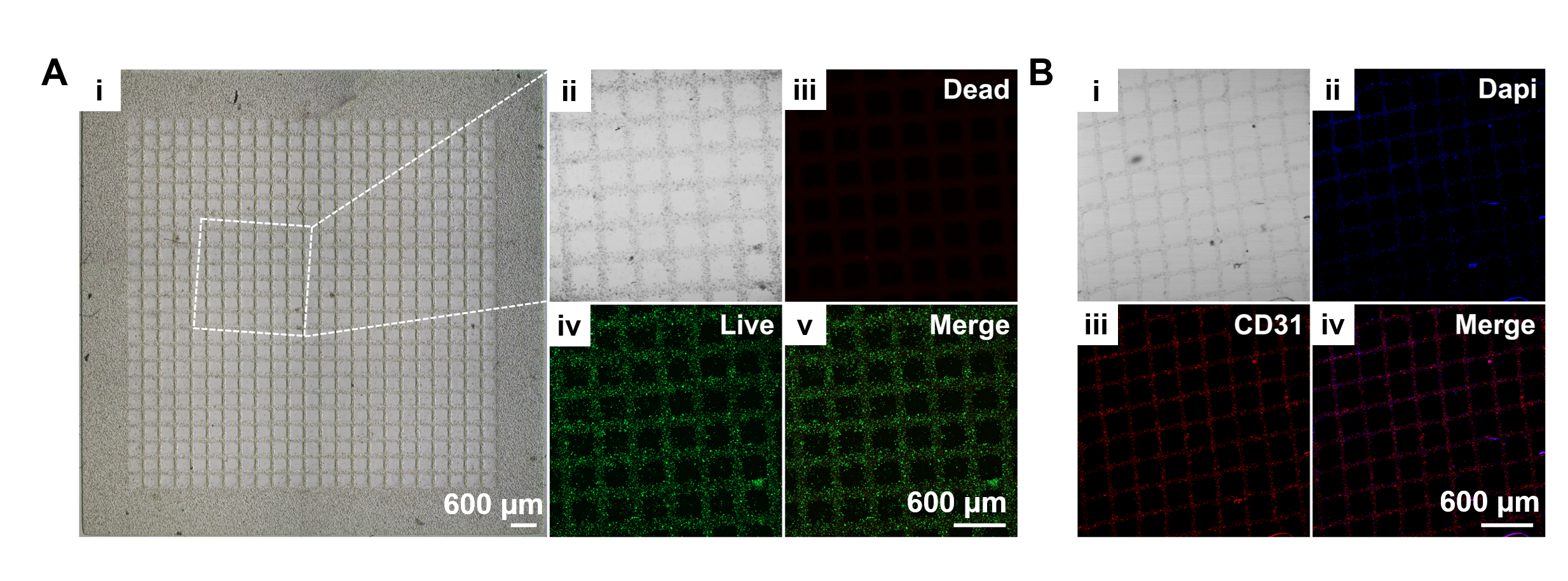
*

***Figure S12.*** *Viability and function of vascular-inductive HUVECs embedded in the waffle-inspired GelMA network of the S-300 “lock” component.* ***(A)*** *Bright-field (i & ii) and projections of 3D confocal stacks (iii-v) of embedded HUVEC cells after fluorescent live/dead staining* ***(B)*** *Bright-field (i) and projections of 3D confocal stacks (ii-iv) of embedded HUVEC cells after red immunofluorescent staining of CD31 and DAPI staining of cellular nuclei. Scale bars: 600 μm.*

***Figure S13.*** *In vitro characterization of endothelial sprouting from HUVECs embedded in S-300 GelMA “lock” components fabricated from different concentrations of GelMA prepolymers. Samples were cultured in vitro for 2 days and assessed with fluorescent live/dead staining followed by optical microscopy (left panel) and confocal microscopy (right panel). White arrows indicated endothelial sprouting from embedded HUVECs showing formation of more mature capillary-like network when GelMA at lower concentration (5% or 7.5% (w/v)) was used for fabrication of S-300 “lock” component. Scale bar representing 500 μm applies to both optical images and fluorescent images.*

**
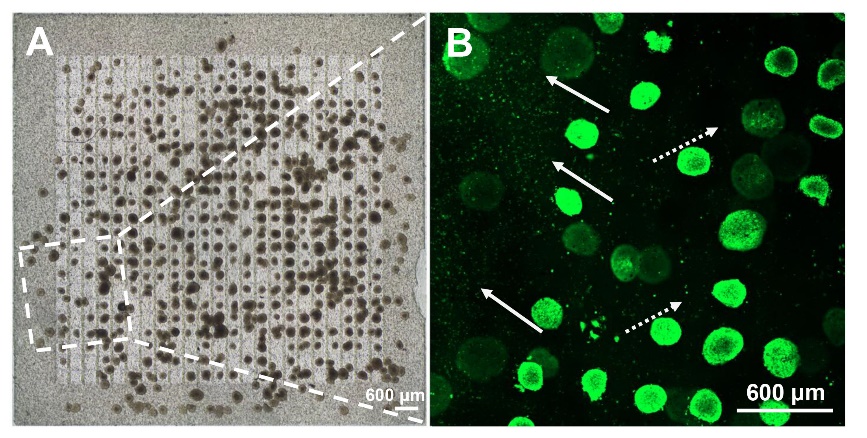
**

***Figure S14.*** *Characterization of cellular viability of co-laden INS-1E and HUVECs in S-300 WIM device using fluorescent live/dead staining followed by confocal microscopy.* ***(A)*** *Bright-field image of S-300 WIM device and* ***(B)*** *3D projection of confocal stacks of co-laden INS-1E microtissue and HUVECs near the outer edge of the device. A higher density of single live cells was detected in the outer edge of device (solid white arrow) which is on the left-hand side of the confocal image while live cells at a lower density (dotted white arrow) was shown in the waffle-inspired GelMA network region within the interior of device, which is on the right-hand side of the confocal image. Scale bars: 600 μm.*
